# Targeting Metabolic Adaptation of Colorectal Cancer with Vanadium‐Doped Nanosystem to Enhance Chemotherapy and Immunotherapy

**DOI:** 10.1002/advs.202409329

**Published:** 2024-12-30

**Authors:** Qian Cheng, Yuzhe Chen, Danyi Zou, Qilin Li, Xiaolei Shi, Qushuhua Qin, Miaodeng Liu, Lin Wang, Zheng Wang

**Affiliations:** ^1^ Research Center for Tissue Engineering and Regenerative Medicine Union Hospital Tongji Medical College Huazhong University of Science and Technology Wuhan 430022 China; ^2^ Department of Clinical Laboratory Union Hospital Tongji Medical College Huazhong University of Science and Technology Wuhan 430022 China; ^3^ Hubei Key Laboratory of Regenerative Medicine and Multi‐disciplinary Translational Research Wuhan 430022 China; ^4^ Hubei Provincial Engineering Research Center of Clinical Laboratory and Active Health Smart Equipment Wuhan 430022 China; ^5^ Department of Gastrointestinal Surgery Union Hospital Tongji Medical College Huazhong University of Science and Technology Wuhan 430022 China

**Keywords:** enhancing chemotherapy and immunotherapy, glucose and glutamine, metabolic reprogramming, nanosystem, therapy resistance

## Abstract

The anti‐tumor efficacy of current pharmacotherapy is severely hampered due to the adaptive evolution of tumors, urgently needing effective therapeutic strategies capable of breaking such adaptability. Metabolic reprogramming, as an adaptive survival mechanism, is closely related to therapy resistance of tumors. Colorectal cancer (CRC) cells exhibit a high energy dependency that is sustained by an adaptive metabolic conversion between glucose and glutamine, helping tumor cells to withstand nutrient‐deficient microenvironments and various treatments. We discover that transition metal vanadium (V) effectively inhibits glucose metabolism in CRC and synergizes with glutaminase inhibitors (BPTES) to disrupt CRC's energy dependency. Thus, a dual energy metabolism suppression nanosystem (VSi‐BP@HA) is engineered by loading BPTES into V‐doped hollow mesoporous silica nanoparticles. This nanosystem effectively dampens CRC energy metabolism, eradicating 33% of tumors in mice. Strikingly, the cell biological and preclinical model datasets provide compelling evidence showing that VSi‐BP@HA not only reverses CRC cells chemo‐resistance but also drastically potentiates anti‐PD1 immunotherapy. Therefore, this nanosystem provides not only a promising approach to suppress CRC, but also a potential adjunct tool for enhancing chemotherapy and immunotherapy.

## Graphical abstract

Based on the metabolic characteristics of colorectal cancer (CRC), researchers have developed a dual energy metabolism suppression nanosystem (VSi‐BP@HA) for enhanced CRC therapy. This nanosystem, which is based on vanadium (V)‐doped silica nanoparticles loading with glutamine metabolic inhibitors, effectively blocks glucose and glutamine metabolism in CRC cells, improving their response to chemotherapy and immunotherapy.

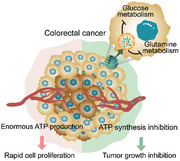

## 1 Introduction

Colorectal cancer (CRC) is the third most commonly diagnosed and the second most fatal cancer worldwide, posing a serious health threat and social burden.^[^ ^1^ ^]^ Currently, surgery, pharmacotherapy, and radiotherapy are the main clinical treatments for CRC. For metastasis and recurrence after surgery and some unresectable tumors, systemic pharmacotherapy is an essential treatment. Unfortunately, current pharmacotherapy strategies, whether traditional chemotherapy or emerging immunotherapy, are severely hampered in their anti‐tumor efficacy due to the adaptive evolution of tumors.^[^ ^2^, ^3^ ^]^ Therefore, an effective and safe pharmacotherapy strategy is urgently needed to break evolutionary adaptability of CRC and improve antitumor therapy efficacy.

**Scheme 1:**
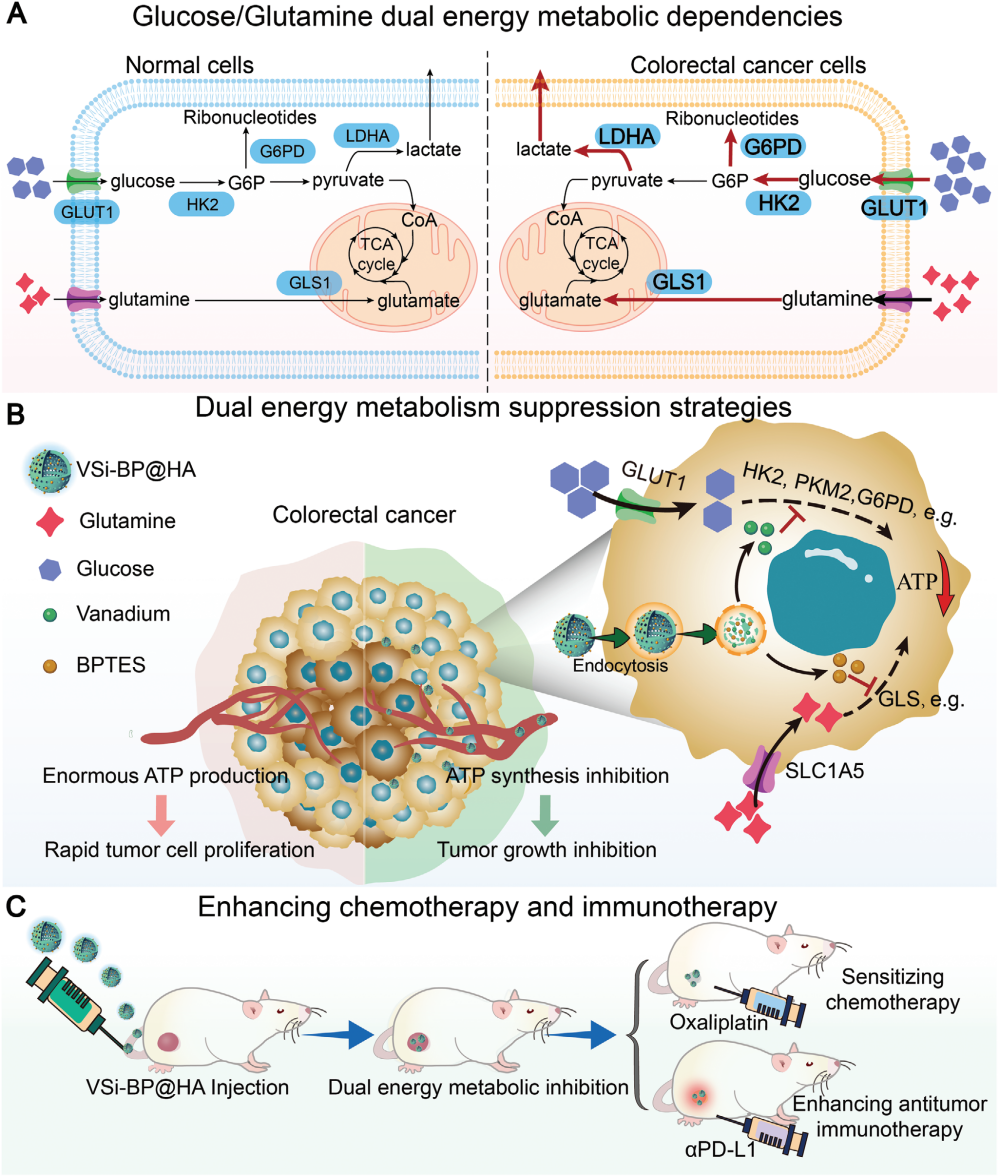
Schematic illustration of the metabolic characteristics of CRC and the working mechanism of VSi‐BP@HA. A) Dual‐energy metabolic dependence of glucose and glutamine in CRC cells. B) The tumor microenvironment (TME)‐responsive dual energy metabolism suppression nanosystem VSi‐BP@HA for specifically killing colorectal tumor cells. C) VSi‐BP@HA sensitizes chemotherapy and immunotherapy.

Metabolic reprogramming, a hallmark of tumors, is closely related to progression, adaptive evolution, and therapy resistance of tumors.^[^ ^4^, ^5^, ^6^ ^]^ To meet augmented bioenergetic requirements for rapid proliferation and cope with varied stresses of existence, tumor cells pathologically evolve various metabolic adaptations to support malignant progression.^[^ ^7^ ^]^ The metabolic adaptation of tumors promotes tumor cells survival under various stress states. For instance, the metabolic adaptive shifts occurring within mitochondria of tumor cells can upregulate the expression of PD‐L1 and TGF‐*β*, thereby contributing to tumor resistance to therapy.^[^ ^8^, ^9^, ^10^ ^]^ However, such a metabolic high demand for nutrients also makes tumor cells rather vulnerable, potentially providing a therapeutic opportunity against tumors.^[^ ^11^ ^]^ Indeed, targeting tumor‐specific energy metabolic vulnerability is emerging as a promising approach for suppressing tumor growth and improving antitumor therapy efficacy.^[^ ^12^ ^]^ Glucose and glutamine are the principal nutrients for tumor cells, and a variety of tumors are reportedly dependent on these two nutrients for proliferation.^[^ ^5^, ^6^, ^13^, ^14^, ^15^, ^16^, ^17^ ^]^ Here, we further confirmed that CRC has an unusually elevated metabolic demand for glucose and glutamine, suggesting that inhibition of glucose or glutamine metabolism could be a promising therapeutic strategy for CRC (**Scheme** **1**A). However, previous reports and our research revealed that solely limiting the metabolism of either glucose or glutamine did not produce desired anti‐CRC effects.^[^ ^13^, ^18^, ^19^ ^]^ Despite being initially effective, this strategy gradually lost its therapeutic power, primarily due to CRC cells’ metabolic re‐adaptability. Once glucose metabolism was restricted, glutamine metabolism was kicked in to compensatorily allow CRC cells to survive under harsh starvation conditions, and vice versa.^[^ ^18^, ^19^, ^20^, ^21^ ^]^ To completely eradicate CRC, overcoming this intractable metabolic adaptation is imperative, which would necessitate development of a drug system that can simultaneously and effectively block the dual metabolic pathways of CRC cells.

Nanomedicines have garnered substantial attention in antitumor therapy due to their unique ability to optimize pharmacokinetics of antitumor agents and simultaneously deliver multiple therapeutic agents with diverse characteristics directly to tumors.^[^ ^22^, ^23^ ^]^ However, when multiple agents are co‐loaded onto one nano‐platform, their disparities in solubility, lipophilicity, charges, and other properties inevitably result in a surge in the complexity and challenges associated with preparation processes.^[^ ^23^ ^]^ Transition metals, due to their distinct electronic structure and chemical properties, can interact with biomolecules, possibly regulating metabolic processes of organisms.^[^ ^24^, ^25^, ^26^ ^]^ An increasing number of studies have been focused on the utilization of transition metal‐doped nanoparticles in tumor treatment, such as Fe, Cu, Mn, Zn, Ag, and Pt.^[^ ^27^, ^28^, ^29^ ^]^ Aside from their inherent anti‐tumor activity, transition metal‐doped nanoparticles also exhibit excellent drug‐loading capacity, which not only helps to reduce the complexity of integrated nanosystems, but also greatly promotes the effectiveness of multi‐modal combined therapy.

As a dietary micronutrient, the transition metal vanadium has numerous medical applications, including anti‐diabetic and anti‐tumor properties.^[^ ^30^, ^31^ ^]^ In this study, we demonstrated that vanadium simultaneously inhibited the activity of multiple glucose metabolism‐related enzymes, effectively blocking the glucose metabolism of CRC cells. Notably, the combination of vanadium and glutaminase inhibitors (bis‐2‐(5‐phenylacetamido‐1,2,4‐thiadiazol‐2‐yl) ethyl sulfide, BPTES) led to strongly synergized antitumor effects. Moreover, compared to normal cells, CRC cells are more sensitive to this combined treatment. Based on this, a dual energy metabolism suppression nanosystem (VSi‐BP@HA) was constructed by loading BPTES into vanadium‐doped hollow mesoporous silica nanoparticles (VSi), which were further modified with hyaluronic acid (HA). Once endocytosed by CRC cells, VSi‐BP@HA was disintegrated and released vanadium ions and BPTES, comprehensively disrupting energy metabolism dependency of CRC cells, and eventually leading to tumor cell apoptosis (Scheme 1B). According to previous reports, metabolic reprogramming provides robust energy support to tumors, enabling their resistance to chemotherapy.^[^ ^11^, ^32^, ^33^, ^34^, ^35^ ^]^ Additionally, adaptive metabolic transformations facilitate the development of immunosuppressive microenvironments, including hypoxia, lactate accumulation, and high TGF‐*β* expression.^[^ ^10^, ^23^, ^36^, ^37^ ^]^ These findings suggest that targeting metabolism would make CRC more vulnerable to chemotherapy and immunotherapy (Scheme 1C). Supporting this notion, VSi‐BP@HA significantly improved CRC's responses to chemotherapy (oxaliplatin, OHP) and immunotherapy (*α*PD‐1). Thus, this dual energy metabolism suppression nanosystem was not only a promising approach to suppress CRC, but also a potential tool for improving standard‐of‐care therapies.

## 2 Results and Discussion

### 2.1 Glucose and Glutamine Metabolic Dependence and Metabolic Adaptability in CRC

**Figure 1:**
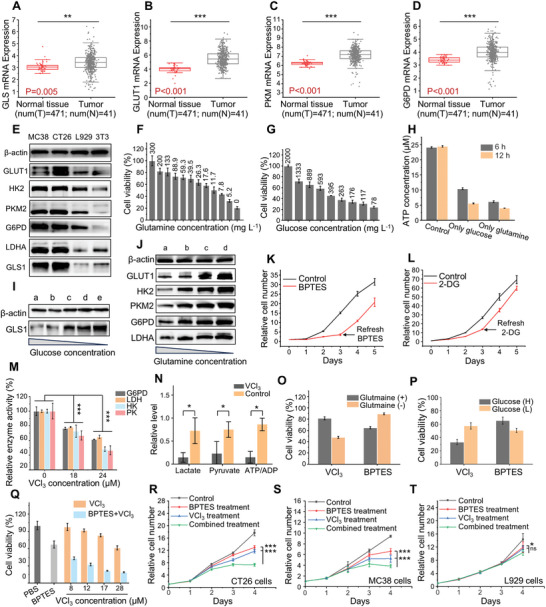
Dual‐energy metabolic dependence and metabolic adaptability in CRC. A‐D) Relative expression levels of A) GLS, B) GLUT1, C) PKM, D) G6PD mRNA from microarray analysis (normalized log2 ratios) of primary tumor samples and adjacent normal tissue from patients with colorectal tumors (the Cancer Genome Atlas (TCGA) database, https://genome‐cancer.ucsc.edu). E) Representative Western blot images of glucose metabolism‐related enzymes (GLUT1, HK2, PKM2, G6PD, LDHA) and GLS1 expression in colorectal tumor cell lines (MC38, CT26) and normal cell lines (L929, 3T3). F) Cell viability of CT26 cells cultured in glutamine‐deficient mediums containing 2000 mg L^−1^ glucose and different concentrations of glutamine (*n* = 4). G) Cell viability of CT26 cells cultured in glucose‐deficient mediums 300 mg L^−1^ glutamine and different concentrations of glucose (*n* = 4). H) The ATP production of CT26 cells cultured in the mediums containing only glucose or glutamine (*n* = 3). I) Representative Western blot images of GLS1 expression in CT26 tumor cells cultured in mediums containing different concentrations of glucose. (a: 4500 mg L^−1^; b: 3000 mg L^−1^; c: 2000 mg L^−1^; d: 1333 mg L^−1^; e: 889mg L^−1^;). J) Representative Western blot images of glucose metabolism‐related enzymes (GLUT1, HK2, PKM2, G6PD, LDHA) expression in CT26 tumor cells cultured in mediums containing different concentrations of glutamine. (a, 450 mg L^−1^; b, 300 mg L^−1^; c, 88.9 mg L^−1^; d, 26.3 mg L^−1^;). K) Relative proliferation of CT26 tumor cells treated long‐term with BPTES (4 µM) or untreated (*n* = 4). As indicated BPTES was refreshed on the third day of the experiment. L) Relative proliferation of CT26 tumor cells treated long‐term with 2‐DG (2 mM) or untreated (*n* = 4). As indicated 2‐DG was refreshed on the third day of the experiment. M) The enzyme activity of G6PD, LDHA, HK2, PKM2 in VCl_3_‐treated CT26 tumor cells (*n* = 4). N) Measurement of glucose metabolites in VCl_3_‐treated CT26 tumor cells (*n* = 4). O,P) Effect of VCl_3_ and BPTES on the proliferation of CT26 cells in varying nutritional conditions (*n* = 8). Viability of CT26 cells treated with 15 µM VCl_3_ or 4 µM BPTES in a glutamine‐deficient culture condition for 2 days (O, “(+)” and “(‐)” represent containing 300 mg L^−1^ glutamine and no glutamine, respectively). Viability of CT26 cells treated with 40 µM VCl_3_ or 2.6 µM BPTES in a glucose‐deficient culture condition for 2 days (P, “(H)” and “(L)” represent containing 2000 mg L^−1^ glucose and 300 mg L^−1^ glucose, respectively). Q) Synergistic effect of BPTES and VCl_3_ on killing CT26 cells (*n* = 3). Cells were treated with 2 µM BPTES and different concentrations of VCl_3_. R‐T) Effect of VCl_3_, BPTES, and combined treatment on R) CT26 cells, S) MC38 cells, T) L929 cells (BPTES: 1 µM; VCl_3_: 12 µM; *n* = 8). Data were represented as mean values ± SD. and statistically calculated by the two‐tailed unpaired student's t‐test (in A to D, and N) and one‐way ANOVA followed by Bonferroni's test (in M, and R to T). *p < 0.05, **p < 0.01, ***p < 0.001. GLS1, glutaminase 1; GLUT1, glucose transporter 1; PKM2, pyruvate kinase isozyme type M2; HK2, hexokinase 2; G6PD, glucose‐6‐phosphate dehydrogenase; LDHA, lactate dehydrogenase A.

Tumor cells can increase nutrient uptake and catabolism by regulating the expression of related enzymes, allowing them to produce a large amount of adenosine triphosphate (ATP) supporting rapid proliferation.^[^ ^4^ ^]^ To investigate whether the expression of energy metabolism‐related genes changes in human CRC, we compared the expression of related genes in colorectal tumors and their adjacent normal tissues using the Cancer Genome Atlas (TCGA) database. Bioinformatics analysis of the representative dataset of colorectal tumors showed that genes related to glucose metabolism (GLUT1, PKM, G6PD, and LDHA) and glutamine metabolism (GLS) were expressed at higher levels in tumor tissues than in adjacent normal tissues (**Figure** **1**A–D; Figure S1, Supporting Information). Consistent with the above findings, the expression of glucose metabolism‐related enzymes (GLUT1, HK2, PKM2, G6PD, and LDHA) and GLS1 was higher in CRC cell lines (CT26 and MC38) compared to normal cell lines (L929 mouse fibroblasts; 3T3 mouse fibroblasts) (Figure 1E; Figure S2, Supporting Information). These results indicate that CRC cells are pathologically featured with an increased demand for glucose and glutamine. Furthermore, to clarify the dependence of CRC cell proliferation on glucose and glutamine metabolism, CRC cells were cultured in glucose‐deficient or glutamine‐deficient mediums, respectively. The proliferation of both CRC cell lines (CT26 and MC38) was positively correlated with glucose or glutamine concentrations in the medium (Figure 1F,G; Figure S3, Supporting Information). In addition, the unavailability of glucose and glutamine in the medium greatly affected ATP production in CT26 cells (Figure 1H) and MC38 cells (Figure S4, Supporting Information). These results suggest that CRC highly depends on the metabolism of glucose and glutamine.

It is worth noting that tumor cells frequently adapt their metabolic phenotypes to tumor microenvironment (TME), which would directly affect the efficacy of tumor metabolic targeting therapy.^[^ ^38^ ^]^ Therefore, we then investigated the levels of metabolic enzyme expression in different nutrient supply environments. As the concentration of either glucose or glutamine in the medium decreased, CT26 cells correspondingly increased GLS1's expression (Figure 1I; Figure S5, Supporting Information) or glucose metabolism‐related enzymes expression (Figure 1J; Figure S6, Supporting Information). This indicates that the deficiency of one nutrient (glucose or glutamine) in the colorectal TME would induce compensatory upregulation of another metabolic pathway to maintain cell proliferation, consistent with the reported metabolic adaptation and metabolic reprogramming capabilities of CRC cells.^[^ ^20^, ^21^ ^]^ Subsequently, we conducted long‐term proliferation experiments in vitro with a GLS1 inhibitor (BPTES) or a glycolysis inhibitor (2‐DG, 2‐Deoxy‐D‐glucose). The results showed that CRC cells (CT26 and MC38) re‐established their baseline proliferation rate at late stages of long‐term treatment with BPTES or 2‐DG, implying that metabolic adaptation compromises the therapeutic effect of blocking a single metabolic pathway (Figure 1K,L; Figure S7, Supporting Information). Therefore, to counteract CRC metabolic adaptation and elevate anti‐CRC efficacy, an integrated dual‐metabolic inhibition nanosystem would be required. However, when multiple inhibitors are co‐loaded onto one nanosystem, their differences in solubility, charges, lipophilicity, and other properties would increase the complexity and difficulty of preparation. Given the ability of transition metal ions to regulate biological metabolism, they might be key to overcoming the challenges during preparation.

### 2.2 Bioactivity of Transition Metal Vanadium and its Combination with BPTES

As previously reported, the transition metal vanadium can affect the glucose metabolism of organisms.^[^ ^39^ ^]^ To determine the specific effects of vanadium on glucose metabolism in CT26 cells, we measured related enzymes activity as well as intermediate product content. The findings revealed that VCl_3_ treatment significantly reduced the activity of glucose metabolism‐related enzymes (G6PD, LDH, HK, and PK) (Figure 1M) and hampered the production of glucose metabolic intermediates (lactate, pyruvate, and ATP) (Figure 1N). These results suggest that vanadium can inhibit the glucose metabolism pathway in tumor cells and may achieve specific and effective inhibitory effects on CRC when combined with GLS1 inhibitor (BPTES). To test this notion, we first investigated the effect of BPTES and VCl_3_ on the proliferation of CRC cells (CT26 and MC38) and normal cells (3T3 and L929), respectively. CRC cells were more sensitive to BPTES and VCl_3_, with lower IC50 values than normal cells (Figure S8, Supporting Information), presumably due to heightened metabolic demands in rapidly proliferating tumor cells. Subsequently, we investigated their inhibitory effects on CRC cells in different nutritional conditions. In the case of glutamine deficiency, CRC cells primarily relied on glucose metabolism, thereby reducing the inhibitory effect of BPTES on proliferation and rendering tumor cells more susceptible to VCl_3_ (Figure 1O). In contrast, tumor cells treated with BPTES in the case of glucose deficiency produced the opposite result because tumor cells primarily relied on glutamine metabolism (Figure 1P). It was demonstrated that the inhibitory effect of VCl_3_ and BPTES on cellular growth was positively correlated with the dependence of tumor cells on glucose and glutamine metabolism. According to Jin's formula,^[^ ^40^ ^]^ all combination index values of BPTES and VCl_3_ combined treatment were greater than 1.15, indicating a synergistic effect between VCl_3_ and BPTES, consistent with previous speculation (Figure 1Q; Figure S9A,B, Supporting Information). Subsequently, the inhibitory effect of the BPTES and VCl_3_ combined treatment on cell proliferation was investigated (Figure 1R–T; Figure S10, Supporting Information). The combined treatment inhibited CRC cell proliferation by 2.40 folds (CT26) and 2.45 folds (MC38) on the 5th day. However, the inhibition effect on normal cells was relatively weak, 1.38 folds for L929 (mouse fibroblasts), 1.22 folds for NCM460 (human colon epithelial cells), 1.27 folds for HUVEC (Human umbilical vein endothelial cells), and 1.72 folds for 3T3 (mouse fibroblasts), respectively, which could be attributed to the difference observed in metabolic requirements of normal cells and CRC cells. Furthermore, we discovered that inhibiting either glucose or glutamine metabolism had a significantly weaker inhibitory effect on CRC cell proliferation than inhibiting both glucose and glutamine metabolism simultaneously (Figure 1R–T).

Collectively, the combination treatment of vanadium and GLS1 inhibitor (BPTES) can specifically and efficiently inhibit the energy metabolism in CRC cells, thereby inhibiting their proliferation.

### 2.3 Preparation and Characterization of VSi‐BP@HA

**Figure 2:**
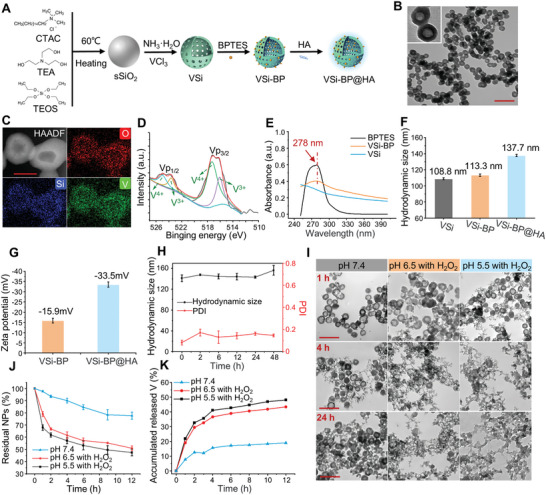
Synthesis and characterization of VSi‐BP@HA. A) Preparation of VSi‐BP@HA. B) The representative TEM image of VSi. Scale bar: 200 nm. C) The HAADF‐STEM image and corresponding element mapping of VSi nanoparticles. Scale bar: 50 nm. D) XPS spectra of V 2p in VSi. E) UV–vis absorption spectra of BPTES, VSi, VSi‐BP. F) Hydrodynamic particle size of Si, VSi‐BP, VSi‐BP@HA (*n* = 3). G) Zeta potential of VSi‐BP, VSi‐BP@HA (*n* = 3). H) Hydrodynamic size and polydispersity index (PDI) of VSi‐BP@HA immersed in PBS buffer for different times (*n* = 3). I) The representative TEM images of VSi‐BP@HA after incubation with different buffers (pH 7.4, pH 6.5 with 100 µM H_2_O_2_, pH 5.5 with 100 µM H_2_O_2_). Scale bar: 200 nm. J) The degradation curve of VSi@HA determined by UV–vis absorption (pH 7.4, pH 6.5 with 100 µM H_2_O_2_, pH 5.5 with 100 µM H_2_O_2_; *n* = 3). K) The release curve of V detected by ICP‐MS in different conditions (pH 7.4, pH 6.5 with 100 µM H_2_O_2_, pH 5.5 with 100 µM H_2_O_2_; *n* = 3). NPs represent nanoparticles. Data are shown as mean ± SD. CTAC, cetyltrimethylammonium chloride; TEA, triethanolamine; TEOS, tetraethyl orthosilicate; BPTES, bis‐2‐(5‐phenylacetamido‐1,2,4‐thiadiazol‐2‐yl) ethyl Sulfide; HA, hyaluronic acid.

Based on the above results, we developed an integrated nanoplatform (VSi‐BP@HA) that could simultaneously release vanadium and GLS1 inhibitors (BPTES) in response to TME, allowing for dual inhibition of tumor energy metabolism. The synthesis steps of VSi‐BP@HA are depicted in **Figure** **2**A. First, monodisperse solid silicon spheres (sSiO_2_) were prepared according to a previously reported experimental method (Figure S11A,B, Supporting Information).^[^ ^25^ ^]^ Subsequently, sSiO_2_ was dispersed in a weakly alkaline solution containing vanadium chloride under heating, causing slow degradation inside sSiO_2_ and gradually forming hollow V‐doped silica nanoparticles (VSi) (Figure 2B). Additionally, sSiO_2_ was also dispersed in a slightly alkaline solution under heating to obtain hollow sSiO_2_ (hSiO_2_, Figure S11C, Supporting Information). High‐angle annular dark‐field scanning transmission electron microscopy (HAADF‐STEM) and corresponding elemental mapping confirmed that V, Si, and O elements were uniformly distributed within VSi nanoparticles (Figure 2C). The vanadium content in VSi was determined to be 7.0% using inductively coupled plasma mass spectrometry (ICP‐MS). The chemical state of the V element in VSi was investigated using X‐ray photoelectron spectroscopy (XPS) (Figure 2D). The high‐resolution XPS spectra of V 2p exhibited two characteristic sets of peaks (516.08 eV, 523.62 eV, and 517.28 eV, 524.94 eV, based on the 284.8 eV C 1s reference), corresponding to the binding energies of V^4+^ and V^3+^, respectively.^[^ ^41^, ^42^ ^]^ BPTES was successfully loaded into VSi, as evidenced by the UV‐vis absorption spectrum, where VSi‐BP exhibited a characteristic absorption peak matching BPTES at ≈278 nm (Figure 2E). By further comparing the absorbance of the supernatant and the initial BPTES at 278 nm, the loading efficiency and loading content of BPTES in VSi‐BP were calculated to be ≈94.68% and 13.26%, respectively (Figure S12, Supporting Information). Finally, to improve the dispersibility and stability of nanoparticles under physiological conditions, HA was modified onto the surface of VSi‐BP, forming VSi‐BP@HA. Compared with unmodified nanoparticles (VSi‐BP), HA‐modified nanoparticles (VSi‐BP@HA) featured a larger hydrodynamic particle size (137.7 nm) and a higher negative surface potential (−33.5 mV) (Figure 2F,G). When VSi‐BP@HA was dispersed in PBS buffer for 48 h, there was no discernible change in hydrodynamic particle size and no signs of aggregation or sedimentation, suggesting the favorable stability of VSi‐BP@HA in physiological conditions (Figure 2H; Figure S13, Supporting Information).

### 2.4 Degradation of VSi‐BP@HA in Response to TME

Typically, the V─O bond would be broken under the action of H^+^, and V4^+^/V3^+^ was easily oxidized in acidic high‐H_2_O_2_ TME.^[^ ^43^ ^]^ Therefore, we speculated that TME would accelerate the structure collapse and degradation of VSi‐BP@HA, resulting in the release of vanadium ions and BPTES. To verify this notion, VSi‐BP@HA was dispersed in different solutions (pH 7.4, pH 6.5 with H_2_O_2_, pH 5.5 with H_2_O_2_) to evaluate its degradation performance using transmission electron microscopy (TEM) (Figure 2I). After a 24 h incubation in the neutral buffer solution, most of the nanoparticles maintained their morphological integrity. In contrast, VSi‐BP@HA incubated in acidic buffers (pH 5.5 and pH 6.5) containing H_2_O_2_ disintegrated significantly after 4 h, with only residual VSi‐BP@HA fragments after 24 h. Further combining the degradation curve of VSi@HA determined by UV–vis absorption (Figure 2J; Figure S14, Supporting Information) and the release curve of V detected by ICP‐MS (Figure 2K), it could be assumed that VSi‐BP@HA would undergo degradation and release vanadium ions and BPTES in the acidic microenvironment (containing H_2_O_2_). In addition, VSi‐BP@HA nanoparticles only caused ≈2% hemolysis of mouse red blood cells at a high concentration of 250 mg L^−1^, indicating a good hemocompatibility of VSi‐BP@HA (Figure S15, Supporting Information). These in vitro experimental results indicate the high biocompatibility and specific responses to TME of the synthesized VSi‐BP@HA.

### 2.5 Inhibition of Tumor Cell Metabolism and Proliferation by VSi‐BP@HA in vitro

**Figure 3:**
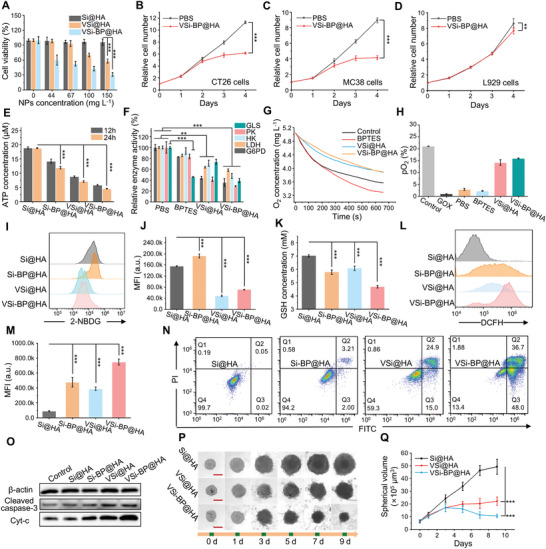
Mechanism of CRC inhibition by VSi‐BP@HA in vitro. A) Cell viability of CT26 cells incubated with different nanoparticles for 24 h (*n* = 8). B‐D) Effect of VSi‐BP@HA treatment on the proliferation of B) CT26 tumor cells, C) MC38 tumor cells, and D) L929 cells, *n* = 8. E) ATP detection in CT26 cells exposed to different nanoparticles for 12 and 24 h (40 µg mL^−1^, *n* = 3). F) The enzyme activity of G6PD, LDH, HK, PK, and GLS in CT26 cells treated with BPTES (8 µM), VSi@HA (40 µg mL^−1^), and VSi‐BP@HA (40 µg mL^−1^) for 24 h (*n* = 3). G) Change of Oxygen content in mediums under the sealed condition. Before detection, CT26 cells were treated with BPTES (4 µM), VSi@HA (40 µg mL^−1^) or VSi‐BP@HA (40 µg mL^−1^) for 4h. H) Partial pressure of oxygen (pO_2_) in CT26 cells after different treatments under the sealed condition for 8h (*n* = 3). I,J) Detection of glucose uptake of CT26 cells after treatment with various nanoparticles (60 µg mL^−1^) for 24 h via I) flow cytometry and the J) corresponding quantization results (*n* = 3). K) GSH detection of CT26 cells treated with different nanoparticles (30 µg mL^−1^) for 24 h (*n* = 3). L,M) Measurement of ROS in CT26 cells treated with various nanoparticles (40 µg mL^−1^) for 24 h via L) flow cytometry and the M) corresponding quantization results (*n* = 3). N) Evaluation of apoptosis of CT26 incubated with nanoparticles (60 µg mL^−1^) for 24h by flow cytometry. O) Representative Western blot images of apoptosis‐related protein (cytochrome c, cleaved caspase‐3) expression in CT26 cells treated with nanoparticles (60 µg mL^−1^) for 24 h. P,Q) Effect of nanoparticles (30 µg mL^−1^) on the proliferation of CT26 tumor spheroid. The medium containing nanoparticles was refreshed every 2 days, scale bar: 200 µm, *n* = 8. Data were represented as mean values ± SD. and statistically calculated by the two‐tailed unpaired student's t‐test (in B‐D) and one‐way ANOVA followed by Bonferroni's test (in A, E, F, J, K, M, Q); *p < 0.05, **p < 0.01, ***p < 0.001. GLS, glutaminase; PK, pyruvate kinase; HK, hexokinase; G6PD, glucose‐6‐phosphate dehydrogenase; LDH, lactate dehydrogenase.

Subsequently, the inhibitory capacity and mechanism of VSi‐BP@HA on CRC cells were investigated. VSi@HA exhibited a 42% cell‐killing rate at a concentration of 150 mg L^−1^ (**Figure** **3**A), which was attributed to the release of vanadium ions from VSi@HA degradation. In addition, VSi‐BP@HA treatment elicited the strongest inhibition on CT26 cell viability, which was superior to VSi@HA treatment, presumably due to the previously verified synergistic metabolic inhibitory effects of vanadium and BPTES (Figure 1Q; Figure S9, Supporting Information). MC38 cells treated with VSi‐BP@HA produced similar results (Figure S16, Supporting Information). Following that, the inhibitory effects of VSi‐BP@HA on CRC cell and normal cell proliferation were investigated. VSi‐BP@HA treatment inhibited CRC cell proliferation by 1.84 folds (CT26) and 2.17 folds (MC38) on the fifth day (Figure 3B–D; Figure S17, Supporting Information). However, the inhibitory effect on normal cells was relatively weak, 1.12 folds (L929) and 1.21 folds (3T3), respectively, which may be attributed to the metabolic difference between normal cells and CRC cells. To validate the speculation, the effects of VSi‐BP@HA on CRC cell energy metabolism were examined. Si‐BP@HA, VSi@HA, and VSi‐BP@HA all caused ATP synthesis inhibition in CT26 cells (Figure 3E), with VSi‐BP@HA causing the strongest ATP synthesis inhibition. In addition, VSi@HA significantly inhibited the activities of glucose‐related enzymes (G6PD, LDH, HK, and PK), and VSi‐BP@HA simultaneously inhibited the activities of glucose‐related enzymes as well as GLS (Figure 3F). Because aerobic respiration is one of the primary pathways of glucose metabolism, we further investigated the oxygen consumption of tumor cells following various treatments. Under sealed conditions, VSi@HA and VSi‐BP@HA inhibited CT26 cell oxygen consumption in medium, indicating that they restrain aerobic respiration. Notably, BPTES treatment increased cell oxygen consumption, possibly due to compensatory elevated glucose metabolism demand in CT26 cells caused by glutamine metabolism inhibition (Figure 3G). Further investigation into the oxygen partial pressure (pO_2_) in CT26 cells yielded consistent results (Figure 3H). Following that, glucose uptake by tumor cells was assessed using a fluorescent glucose analogue, 2‐(N‐(7‐nitrobenz‐2‐oxa‐1,3‐diazol‐4‐yl)‐amino)‐2‐deoxyglucose (2‐NBDG). VSi@HA significantly inhibited glucose uptake by CT26 cells (Figure 3I,J), which could be attributed to released vanadium ions reducing the activity of enzymes related to glucose metabolism, thereby inhibiting glucose uptake and utilization by CT26 cells. Conversely, Si‐BP@HA promoted glucose uptake due to obstruction of glutamine metabolism by BPTES. Notably, the glucose uptake after VSi‐BP@HA treatment was only slightly higher than after VSi@HA treatment. This could be because vanadium ions synchronously disrupted glucose‐related metabolic enzyme activity although BPTES promoted a compensatory increase in glucose uptake, causing irreversible impairments to glucose metabolic pathways. These results together confirmed that VSi‐BP@HA where V and BPTES were integrated could simultaneously disrupt glutamine and glucose metabolism in CRC cells.

In addition, BPTES, an allosteric inhibitor of GLS1, effectively hinders the catabolic process of glutamine and the supply of glutamate nitrogen sources, affecting the synthesis of glutathione (GSH).^[^ ^13^, ^44^ ^]^ When compared to normal cells, CRC cells exhibit a pro‐oxidative state that leads to intrinsic oxidative stress (Figure S18A,B, Supporting Information). GSH, an essential intracellular reactive oxygen species scavenger (ROS), is critical for maintaining the REDOX balance in tumor cells,^[^ ^45^ ^]^ and its expression in CRC cells is typically higher than in normal cells (Figure S18C, Supporting Information). Si‐BP@HA exhibited a moderate inhibitory effect on GSH synthesis in CT26 tumor cells, resulting in an 18% reduction compared to Si@HA (Figure 3K). Remarkably, VSi@HA also reduced the intracellular GSH level, presumably as a result of GSH being consumed by a transition metal‐reducing process.^[^ ^45^, ^46^ ^]^ Due to the dual effects of BPTES and the transition metal vanadium, VSi‐BP@HA dramatically reduced intracellular GSH (33%). In addition, VSi‐BP@HA treatment significantly increased ROS levels in CT26 cells (Figure 3L,M). Therefore, VSi‐BP@HA could decrease intracellular GSH levels, disrupting the redox balance in CRC cells. Notably, VSi‐BP@HA, containing transition metal element V, could harness the overproduced H_2_O_2_ within TME to catalyze Fenton‐like reactions, generating highly cytotoxic ·OH radicals that kill tumor cells (Figure S19A, Supporting Information). However, the cytotoxic effect would be limited by the catalytic efficiency of nanoparticles and the availability of endogenous H_2_O_2_ (Figure S19B,C, Supporting Information).^[^ ^47^ ^]^

Furthermore, to clarify the mechanism of tumor cell death induced by VSi‐BP@HA, flow cytometry analysis of apoptosis and necrosis was performed. VCl_3_ and VSi@HA treatments induced 34% and 40% cell apoptosis (Figure 3N; Figure S20A, Supporting Information), while the VCl_3_ and BPTES combined treatment and VSi‐BP@HA treatment caused substantial cell apoptosis by 87% and 85%. Furthermore, immunoblot analyses revealed that the treatments of Si‐BP@HA, VSi@HA, and VSi‐BP@HA all increased cytoplasmic cytochrome C (cyt‐c) and cleaved caspase‐3 expression in CT26 cells, with the greatest increase observed in the treatment with VSi‐BP@HA (Figure 3O; Figure S21, Supporting Information). BPTES, VCl_3_, and BPTES+VCl_3_ treated tumor cells exhibited similar results (Figure S20B,C, Supporting Information). These findings suggest that BPTES+VCl_3_ and VSi‐BP@HA mediated energy metabolism disruption and redox imbalance can induce tumor cell apoptosis via the mitochondrial pathway. Subsequently, a 3D tumor spheroid model was utilized to further investigate the inhibitory effect of BPTES, VCl_3_, and nanoparticles on colorectal tumors. BPTES or VCl_3_ treatment only inhibited tumorsphere growth to a limited extent (Figure S22, Supporting Information), whereas BPTES and VCl_3_ combined treatment almost completely eradicated tumorspheres. Consistently, VSi@HA moderately slowed tumor spheroid growth rate (≈55%), while VSi‐BP@HA produced a superior inhibition effect, with an inhibition rate of ≈80% (Figure 3P,Q). Collectively, this evidence suggests that VSi‐BP@HA nanosystem blocks glucose and glutamine metabolism in colorectal tumors, disrupts energy supply and REDOX homeostasis, and ultimately effectively induces CRC cell apoptosis.

### 2.6 In Vivo Biosafety and Antitumor Efficacy Assessment

**Figure 4:**
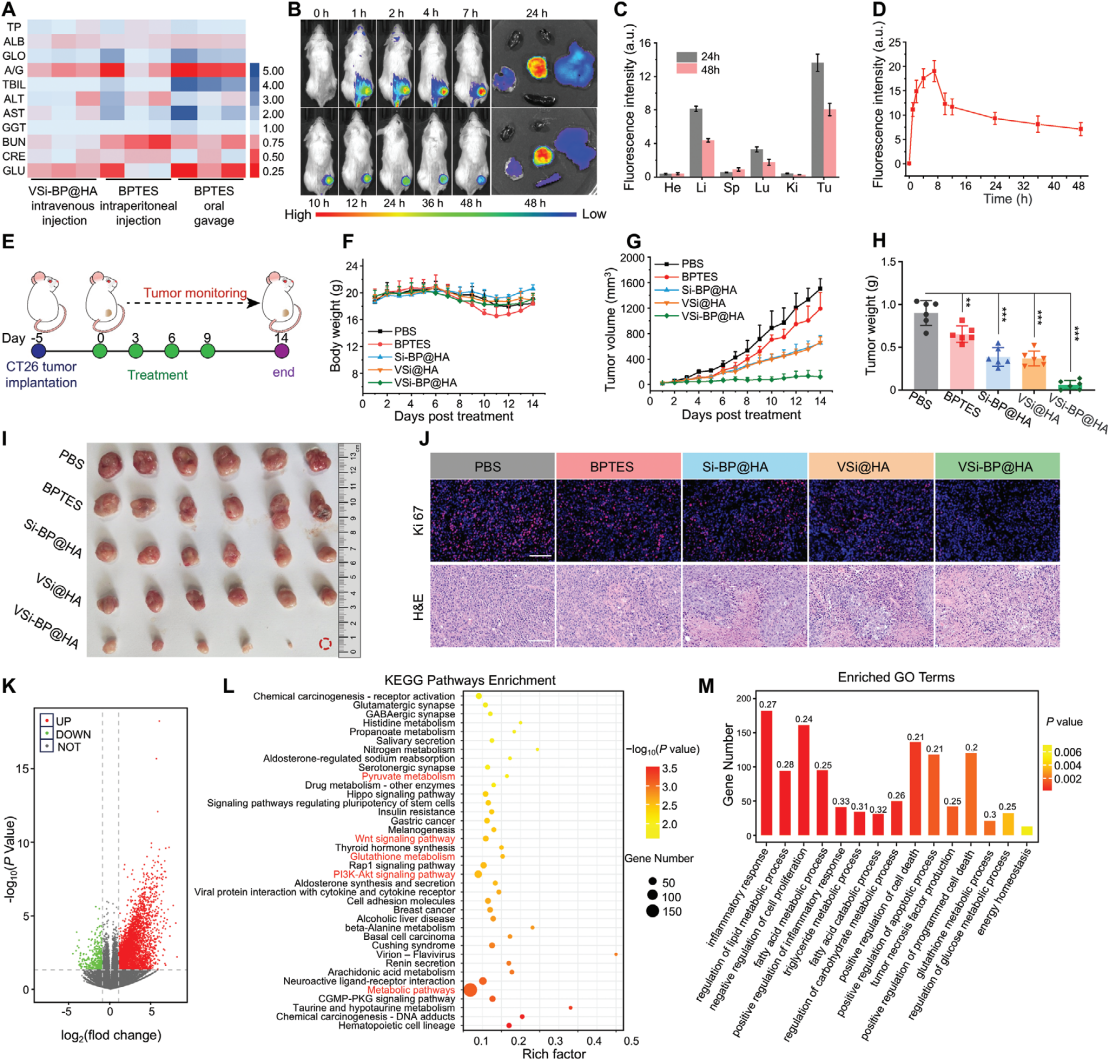
Tumor‐growth inhibition by VSi‐BP@HA nanosystems in vivo. A) Blood biochemistry examination of BALB/C mice undergoing different treatments. VSi‐BP@HA (10 mg kg^−1^) by intravenous injection, free BPTES (5 mg kg^−1^) by intraperitoneal injection and oral gavage. (Naive mice were conducted as control, and all data were processed by normalizing the values of the blank. *n* = 3). B) Representative in vivo fluorescent images of CT26‐tumor‐bearing mice at different times after intravenous injection of VSi‐BP@HA^cy5.5^ (left), and the ex vivo fluorescence images of tumors and major organs (heart, liver, spleen, kidney, lung). C) Corresponding quantitative fluorescent intensity of major organs and tumors (*n* = 4). D) Line graph of fluorescent intensity at tumor site corresponding to B) (*n* = 4). E) Schematic diagram of antitumor therapy for CT26 tumor‐bearing mice. F) Changes in body weight of CT26 tumor‐bearing mice after different treatments (*n* = 6). G) Average growth curves of CT26 tumors following various treatments over 14 days (*n* = 6). H,I) H) weight and I) Photographs of tumors isolated from mice on the 14th day of different treatments (*n* = 6), the red dashed circle represents the complete disappearance of the tumor following VSi‐BP@HA treatment. J) Representative Ki67 immunofluorescence staining and H&E staining images of tumors after 14 days of treatment (Scale bar: 100 µm). K) Volcano plot presenting 1910 differentially expressed genes (31676 total genes) between the control group and VSi‐BP@HA group. L) KEGG enrichment analysis of differentially expressed genes between the control group and VSi‐BP@HA group. M) Significant change pathways induced by VSi‐BP@HA treatment compared to untreated control group from functional enrichment analysis based on the biological process of gene ontology (GO‐BP). Data were represented as mean values ± SD. and statistically calculated by one‐way ANOVA. *p < 0.05, **p < 0.01, ***p < 0.001. TP, total protein; ALB, albumin; GLO, globulin; A/G, albumin/globulin; TBIL, total bilirubin; ALT, alanine aminotransferase; AST, aspartate aminotransferase; GGT, gamma‐glutamyltransferase; CRE, creatinine; GLU, glucose.

Before proceeding to assess the in vivo antitumor efficacy of VSi‐BP@HA, we initially investigated its biosafety. Performing routine blood tests on mouse models, we confirmed that VSi‐BP@HA did not induce acute inflammatory responses (Figure S23A, Supporting Information). In blood chemistry analyses, the alterations in blood chemistry parameters following VSi‐BP@HA treatment were minimal compared to those observed in other treatment groups (**Figure** **4**A; Figure S23B, Supporting Information). The mice treated with free BPTES via intraperitoneal injection or oral gavage suffered hepato‐renal dysfunction, likely due to the high hydrophobicity of BPTES making it susceptible to liver enrichment (Figure S23C, Supporting Information). Subsequently, the tumor targeting and retention properties of VSi‐BP@HA, as well as its biodistribution in vivo, were assessed. First, the cyanine 5.5‐labeled VSi‐BP@HA (VSi‐BP@HA^cy5.5^) was employed. in vivo fluorescence imaging (Figure 4B) and corresponding fluorescence quantification of major organs and tumors (Figure 4C) revealed that VSi‐BP@HA^cy5.5^ exhibited a pronounced enrichment within tumors. This accumulation increased steadily over time, peaking at ≈7 h and remaining high until 48 h post‐injection, indicating that VSi‐BP@HA has favorable tumor targeting and retention capabilities (Figure 4D). We observed a moderate accumulation of the VSi‐BP@HA^cy5.5^ in the liver, with a comparatively negligible accumulation in other organs (Figure 4B,C). Consequently, continuous monitoring of the fluorescent signals in livers was conducted (Figure S24, Supporting Information). We found that VSi‐BP@HA^cy5.5^ was efficiently metabolized within 5 days, suggesting a low risk of long‐term toxicity induced by these nanoparticles. Subsequently, we assessed the metabolic behavior of the VSi‐BP@HA in vivo by quantifying the amount of Si present in various tissues (Figure S25, Supporting Information). The results indicated that the accumulation of VSi‐BP@HA in major organs was low and did not exhibit prolonged retention, suggesting that their potential toxic side effects were weak, consistent with previous results. In addition, the content of Si in the bloodstream (Figure S25D, Supporting Information) decreased slowly over the initial 8 h, subsequently entering a phase of relatively stable levels, with detectable contents persisting for up to 48 h. This indicated that VSi‐BP@HA possessed high stability in blood circulation and prolonged blood retention. The exceptional tumor targeting capacity and favorable biodistribution characteristics exhibited by VSi‐BP@HA are primarily attributed to their possession of appropriate sizes and surface potentials suitable for systemic circulation (Figure 2F,G), as well as their demonstrated superior dispersion and stability in physiological environments (Figure 2H; Figure S13, Supporting Information). These properties collectively facilitate the passive accumulation of VSi‐BP@HA at tumor sites via the enhanced permeability and retention (EPR) effect. Additionally, HA layer on the surface of VSi‐BP@HA can engage in specific binding with the CD44 receptor overexpressed by tumor cells, thereby guiding the active targeting of nanoparticles to tumors.^[^ ^48^, ^49^ ^]^

Then, a CT26 tumor‐bearing mouse model was constructed, and all mice were randomly divided into 5 groups: PBS, BPTES, Si‐BP@HA, VSi@HA, and VSi‐BP@HA. The antitumor therapeutic procedure was depicted in Figure 4E, wherein alterations in body weight and tumor volume of mice were monitored for 14 days after the initial administration. The tumors in the mice injected intraperitoneally with BPTES were only slightly inhibited (Figure 4G; Figure S26, Supporting Information), presumably due to the inability of BPTES to effectively deliver to tumor sites toward producing tumor inhibitory effects. Furthermore, Si‐BP@HA and VSi@HA treatments moderately inhibited tumor growth (30% and 40%, respectively), indicating that while nanoparticles are more effectively enriched at tumor sites, the suppression of tumor cell metabolism via a single pathway is limited. Notably, VSi‐BP@HA exhibited the most potent tumor suppressive effect, with 33% of tumors almost completely eradicated, indicating the potentiality of such a dual energy metabolism inhibitory system against tumors (Figure 4G; Figure S23, Supporting Information). All the mice were euthanized at the end of treatment, the photos (Figure 4I) and weights (Figure 4H) of isolated tumors in each group further confirmed the above observations. The body weight of the mice remained relatively stable after various nanoparticle treatments (Figure 4F). However, the free BPTES‐treated mice experienced a noticeable decrease in body weight on day 8 and gradually returned to normal levels after discontinuation of administration. It is most likely due to the toxic side effects caused by BPTES’ liver enrichment. Meanwhile, no appreciable histological damage was observed in hearts, livers, spleens, lungs, and kidneys (Figure S27, Supporting Information), further indicating that VSi‐BP@HA possesses excellent biosafety. In addition, representative tumor tissue slices stained with hematoxylin and eosin (H&E) revealed that VSi‐BP@HA treatment caused the most dramatic morphological changes in tumor cells and damaged tissue (Figure 4J). Consistently, immunofluorescence staining exhibited the weakest cell proliferation signal (Ki67, Figure 4J) and the greatest cell apoptosis signal (Cleaved caspase‐3, Figure S28, Supporting Information) in VSi‐BP@HA treatment group. Based on the above results, it is demonstrated that this integrated nano‐formulation exhibits significant advantages over antibodies and inhibitors in addressing metabolic adaptability of tumor cells, including excellent tumor targeting efficacy, relatively lower side effects, superior pharmacokinetic profiles, and more pronounced anti‐tumor effects.^[^ ^50^, ^51^ ^]^

To investigate the underlying mechanism of action of VSi‐BP@HA on tumor tissue, transcriptome sequencing analysis of CT26 tumor tissues was performed. The tumor tissues of untreated CT26 tumor‐bearing BALB/c mice served as a control group. As shown by the volcano plot (Figure 4K), a total of 31676 genes were analyzed, and 1910 differentially expressed genes (DEGs) were screened under a threshold with |log2(foldchange)| >2 and p values < 0.05, indicating the significant difference between VSi‐BP@HA and control groups. Then, the Kyoto Encyclopedia of Genes and Genomes (KEGG) pathway enrichment analysis of these DEGs confirmed that VSi‐BP@HA treatment markedly affected metabolism‐related signaling pathways (e.g., metabolic pathways, pyruvate metabolism, glutathione metabolism), and regulated inflammation‐related signaling pathways (e.g., wnt signaling pathway, PI3K‐Akt signaling pathway) (Figure 4L). Furthermore, Gene Ontology (GO) function enrichment analysis was performed to study the associated biological function of these DEGs. The biological processes of cell death and proliferation, as well as the inflammatory response in tumor tissue, were influenced by VSi‐BP@HA treatment (Figure 4M). The expression of the genes associated with “positive regulation of apoptotic process” and “negative regulation of cell proliferation” was up‐regulated (Figure S29, Supporting Information). In addition, some important substance metabolic processes (e.g., lipid, fatty acid, triglyceride, carbohydrate, glutathione, and glucose) in tumor tissues were also affected (Figure 4M). Some genes (e.g. Dcn, Sesn3, Rufy4, Adrd2, et al.) involved in positive regulation of autophagy were upregulated, presumably due to VSi‐BP@HA treatment disrupting the energy metabolic balance and redox homeostasis of tumor tissue (Figure S30, Supporting Information). Collectively, the nanoparticles could disrupt tumor metabolism and induce local inflammation, ultimately suppressing cell proliferation and promoting cell death.

### 2.7 VSi‐BP@HA Sensitized Chemotherapy

**Figure 5:**
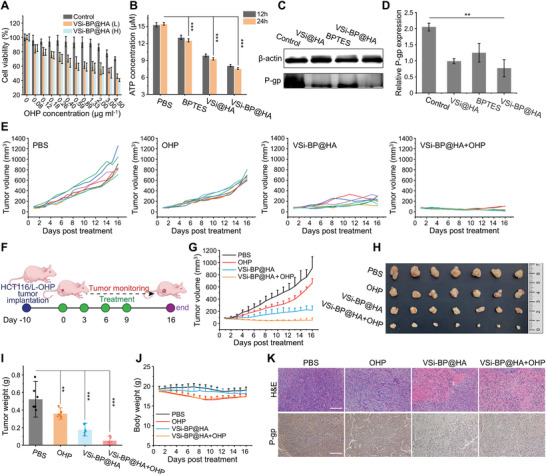
Evaluation of VSi‐BP@HA nanosystem sensitized tumor chemotherapy. A) Cell viability of HCT116/L‐OHP cells grown with VSi‐BP@HA (“L” represents low concentration: 1.73 µg mL^−1^; “H” represents high concentration: 2.6 µg mL^−1^) and different concentrations of OHP for 48h (*n* = 8). B) The ATP production in HCT116/L‐OHP cells incubated with BPTES (8 µM) or different nanoparticles (VSi@HA, VSi‐BP@HA, 40 µg mL^−1^) for 24 h (*n* = 3). C) Representative Western blot images of P‐gp expression in HCT116/L‐OHP cells treated with BPTES (8 µM) or different nanoparticles (VSi@HA, VSi‐BP@HA, 40 µg mL^−1^) for 24 h. D) Semi‐quantitative analysis of P‐gp expression in HCT116/L‐OHP cells with different treatments (n = 3). E) Individual tumor growth curves of HCT116/L‐OHP tumor‐bearing mice after different treatments. F) The schematic diagram of treatment schedule in an OHP‐resistant tumor model (HCT116/L‐OHP tumor‐bearing mice). The mice were randomly divided and injected with PBS, OHP (10 mg kg^−1^, *i.p*.), VSi‐BP@HA (10 mg kg^−1^, *i.v*.), OHP + VSi‐BP@HA every 3 days for 4 times. G) Average tumor growth curves of HCT116/L‐OHP tumor‐bearing mice after different treatments (*n* = 7). H,I) H) Photographs and I) average weight of dissected tumors in different groups (*n* = 7). J) Body weight of HCT116/L‐OHP tumor‐bearing mice receiving different therapies (*n* = 7). K) P‐gp immunohistochemical analysis and H&E staining of tumors after 16 days of treatment. (Scale bar: 100 µm). Data were represented as mean values ± SD. and statistically calculated by one‐way ANOVA (in B and I) and the two‐tailed unpaired student's t‐test (in D). *p < 0.05, **p < 0.01, ***p < 0.001. P‐gp, P‐glycoprotein.

Chemotherapy plays a dominant role in clinical tumor treatment, and chemoresistance is primarily responsible for tumor treatment failure.^[^ ^52^ ^]^ Mounting evidence has suggested that tumor metabolism plays a critical role in the emergence and maintenance of tumor chemoresistance.^[^ ^11^, ^32^ ^]^ Inspired by previous findings, we initially investigated the in vitro toxicity of VSi‐BP@HA combined with oxaliplatin (OHP) on L‐OHP‐resistant human colorectal cancer cell line (HCT116/L‐OHP) to analyze the effect of the dual metabolic inhibition nanosystem on drug sensitivity of drug‐resistant CRC (**Figure** **5**A; Figure S31, Supporting Information). Considering the cytotoxicity of VSi‐BP@HA themselves, low concentrations of VSi‐BP@HA resulted in significantly heightened toxicity of OHP toward HCT116/L‐OHP cells, implying that VSi‐BP@HA has the potential to restore the sensitivity of chemotherapy‐resistant tumors. Overexpression of multidrug resistance (MDR) proteins is one of the crucial mechanisms underlying the development of chemotherapeutic drug resistance (Figure S32, Supporting Information). By recognizing and binding to drug molecules, MDR proteins harness the energy derived from ATP hydrolysis to actively pump these drug molecules out of tumor cells, reducing their effective intracellular concentration and ultimately compromising the efficacy of chemotherapy.^[^ ^53^, ^54^ ^]^ The intracellular ATP concentration holds paramount importance for the function and synthesis of MDR proteins, including p‐glycoprotein (P‐gp).^[^ ^55^, ^56^ ^]^ Thus, we investigated the impact of VSi‐BP@HA on ATP production and MDR protein (taking P‐gp as an example) expression in HCT116/L‐OHP cells. Intriguingly, VSi‐BP@HA significantly suppressed ATP production (Figure 5B) and decreased P‐gp expression in HCT116/L‐OHP cells (Figure 5C,D). These results indicate that VSi‐BP@HA could reduce ATP generation, resulting in downregulation of P‐gp expression and contributing to reversal of drug resistance in HCT116/L‐OHP cells.

Subsequently, we established an HCT116/L‐OHP tumor‐bearing mouse model to investigate the efficacy of nanoparticles in enhancing chemotherapy. To circumvent immune rejection caused by the xenotransplantation of human tumor cells into mice and to eliminate the confounding influence of immune responses on the tumor's response to chemotherapy, we selected immunodeficient nude mice as the experimental animals (Figure 5F). OHP treatment only slightly delayed tumor growth due to HCT116/L‐OHP tumor resistance to OHP (Figure 5E,G). The combined treatment of VSi‐BP@HA and OHP yielded the best anti‐tumor outcome, with a tumor inhibition rate of up to 94% when compared to VSi‐BP@HA or OHP alone, which was attributed to VSi‐BP@HA restoring HCT116/L‐OHP tumor sensitivity to OHP (Figure 5H,I). A decline in the weight of OHP‐treated and VSi‐BP@HA+OHP‐treated groups, followed by a steady recovery after cessation of drug administration on the 9th day (Figure 5J), was probably due to the toxic side effects of OHP on mice. To further decipher the antitumor efficacy and mechanism, histopathologic analysis of excised tumor tissue was performed. H&E and TUNEL staining showed the greatest tumor cell damage and apoptosis signal in the VSi‐BP@HA+OHP‐treated group (Figure 5K; Figure S33, Supporting Information). Furthermore, immunohistochemical staining revealed that VSi‐BP@HA and VSi‐BP@HA+OHP treatments drastically downregulated P‐gp expression in tumor tissues (Figure 5K), consistent with the previous results. Together, these results indicate that VSi‐BP@HA is a potential tool for overcoming chemoresistance in CRC.

### 2.8 VSi‐BP@HA‐Mediated Therapy Combined with Immunotherapy

**Figure 6:**
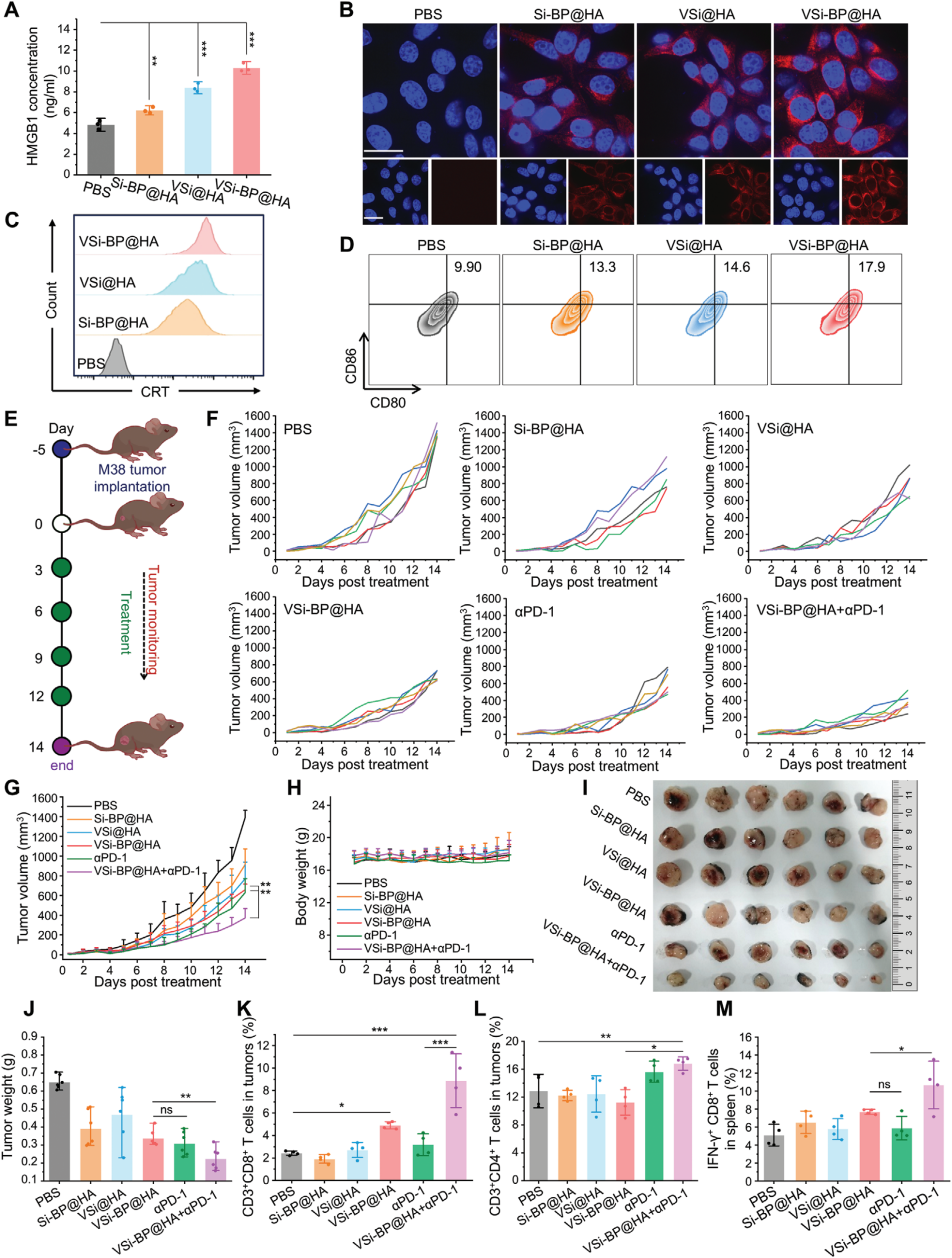
Evaluation of the antitumor therapeutic effect of nanoparticles combined with immune checkpoint inhibitor *α*‐PD1. A) The content of HMGB1 in medium release detected by ELISA (*n* = 3). B) Representative immunofluorescence staining images of calreticulin (CRT) in MC38 tumor cells after different treatments for 12 h (Si‐BP@HA: 80 µg mL^−1^, VSi@HA: 80 µg mL^−1^, VSi‐BP@HA: 80 µg mL^−1^, scale bar: 20 µm). C) Representative flow cytometry image of CRT exposure on the surface of MC38 cells. D) Representative flow cytometry plots of DCs maturation (CD80^+^CD86^+^). E) The treatment plan for MC38 tumor‐bearing mice. The tumor‐bearing mice were randomly grouped and injected with PBS, Si‐BP@HA (10 mg kg^−1^, *i.v*.), VSi@HA (10 mg kg^−1^, *i.v*.), VSi‐BP@HA (10 mg kg^−1^, *i.v*.), *α*PD‐1 (30 µg per mouse, *i.p*.), *α*PD‐1+VSi‐BP@HA every 3 days for 4 times. F) Individual tumor growth curves of MC38 tumor‐bearing mice after different treatments. G) Average tumor growth curves of MC38 tumor‐bearing mice during 14 days of different treatments (*n* = 6). H) Average body weight of mice in different groups (*n* = 6). I,J) I) Photographs and J) average weight of dissected tumors in various treatment groups (*n* = 6). K‐L) Relative abundance of K) CD3^+^CD8^+^ T cells, and L) CD3^+^CD4^+^ T cells in tumors after various treatments as measured by flow cytometry (*n* = 4). M) CD8^+^IFN‐γ^+^ T cells in spleens (gated in CD3^+^, *n* = 4). Data were represented as mean values ± SD. and statistically calculated by one‐way ANOVA. *p < 0.05, **p < 0.01, ***p < 0.001. HMGB1, high mobility group box 1 protein; CRT, calreticulin.

The adverse metabolic competitive advantage in tumor cells and immunosuppressive microenvironment within tumor tissues greatly discount the efficacy of immunotherapy. Consequently, disrupting tumor cell metabolism and reversing the immunosuppressive tumor microenvironment are crucial for improving the therapeutic outcomes of immunotherapy. The above results demonstrated that VSi‐BP@HA treatment elevated ROS levels in CRC cells and disrupted intracellular redox homeostasis (Figure 3K–M). Imbalance of cellular redox homeostasis has been demonstrated to mediate endoplasmic reticulum stress and induce immunogenic cell death (ICD) in tumor cells.^[^ ^57^, ^58^ ^]^ Therefore, we further investigated the ability of VSi‐BP@HA to induce ICD in CRC cells (MC38) by detecting the release of damage‐associated molecular patterns (DAMPs), including high mobility group box 1 (HMGB1) and calreticulin (CRT). HMGB1 is a nuclear protein that is released into the extracellular space when cells are damaged to promote the maturation of DC cells and activate immune responses against tumors. VSi‐BP@HA treatment markedly promoted the release of intracellular HMGB1 into extracellular (**Figure** **6**A). Furthermore, fluorescence microscopy and flow cytometry analysis revealed that the cytomembrane of VSi‐BP@HA treated tumor cells exposed the most abundance of CRT, which acts as an “eat me” signal (Figure 6B,C; Figure S34, Supporting Information). Next, we further explored whether VSi‐BP@HA pretreated MC38 cells could promote dendritic cell (DC) maturation. DC maturation in VSi‐BP@HA pretreated group was the highest of all the groups (Figure 6D; Figure S35, Supporting Information), significantly higher than PBS group. These results suggest that VSi‐BP@HA could induce ICD in CRC cells, thereby boosting anti‐tumor immune responses. Furthermore, previous studies have demonstrated that inhibiting tumor cell energy metabolism in combination with immune checkpoint blockade (ICB) can produce satisfactory therapeutic efficacy.^[^ ^59^, ^60^ ^]^ VSi‐BP@HA was demonstrated to disrupt the metabolic competitive advantage of CRC cells (Figure 3A–J). Therefore, the combination of VSi‐BP@HA with *α*PD‐1 antibody was further conducted in an MC38 tumor‐bearing C57BL/6 mouse model to explore more effective antitumor therapeutic regimens (Figure 6E). The tumor growth curves revealed that both VSi‐BP@HA and *α*PD‐1 exhibited moderate inhibitory effects on tumor growth (Figure 6F,G). However, the combination of VSi‐BP@HA and *α*PD‐1 had the most potent inhibitory effect on tumor growth (Figure 6G,I,J), with a 55% inhibition rate. In addition, the body weight of mice remained stable in all groups throughout the treatment phase, highlighting the safety of the treatment regimens (Figure 6H).

To evaluate the antitumor mechanism of the combination therapy regimen, the levels of T cells in tumors and spleens were detected by flow cytometry at the end of treatment. We observed that VSi‐BP@HA treatment significantly increased the infiltration of CD3^+^CD8^+^ T cells within tumor tissues (Figure 6K), with relative abundances rising from 2.4% in the PBS group to 4.9%. Furthermore, the combination of VSi‐BP@HA with PD‐L1 blockade led to an even more pronounced elevation in the levels of CD3^+^CD4^+^ and CD3^+^CD8^+^ T cells within TME (Figure 6K,L; Figures S36 and S37, Supporting Information). It is noteworthy that the intratumoral infiltration of CD4^+^CD8^+^ T cells in the combination therapy group was 2.79 folds that of the *α*PD‐1 treated group. These results indicated that the combination of VSi‐BP@HA and *α*PD‐1 effectively activated the tumor immune microenvironment. Comparable findings were observed in spleen CD3^+^CD4^+^ and CD3^+^CD8^+^ T cell assessments (Figures S38 and S39, Supporting Information). In addition, the combined therapy synergistically activated CD8^+^ T cells to secrete IFN‐γ in spleen (Figure 6M; Figure S40, Supporting Information). These findings suggest that VSi‐BP@HA combined with *α*PD‐1 treatment provokes a strong immune response, resulting in superior anti‐tumor efficacy. Our developed nanosystem provides an additional option for tumor combination immunotherapy.

## 3 Conclusion

Despite ongoing efforts, the efficacy of tumor pharmacotherapy remains unsatisfactory due to tumor's evolutionary adaptability, necessitating the urgent development of an effective and safe systemic therapeutic modality. Targeting tumor‐specific energy metabolism is a promising approach for tumor treatment due to tumor cells high biosynthetic requirements. In this study, we found that CRC demonstrated unusually high metabolic dependencies for glucose and glutamine, making CRC more vulnerable to glucose and glutamine restrictions. However, the metabolic adaptability of CRC cells rendered single metabolism inhibition inadequate in achieving desired antitumor effects. Previous reports and our research revealed that co‐inhibition of glucose and glutamine metabolism could effectively suppress tumor growth. However, when multiple inhibitors were co‐loaded onto a single nanoplatform, their differences in solubility, lipophilicity, charge, and other properties must be taken into account, inevitably leading to a surge in complexity and preparation challenges.^[^ ^16^, ^61^ ^]^ In this study, we constructed VSi nanoparticles, which not only efficiently inhibited the activity of multiple enzymes of glucose metabolism but also exhibited excellent drug‐loading capacity. To simplify the nanosystem while enhancing the antitumor efficacy of dual metabolic inhibition in vivo, we developed a dual‐energy metabolic inhibition nanosystem (VSi‐BP@HA) by loading BPTES onto VSi nanoparticles and further modifying their surface with HA.

At the tumor site, VSi‐BP@HA decomposed, releasing vanadium ions capable of suppressing glucose metabolism and BPTES effective in inhibiting glutamine metabolism, thereby comprehensively disrupting the energy metabolism homeostasis within CRC cells. The comprehensive in vitro and in vivo results demonstrated that VSi‐BP@HA not only significantly disrupted CRC's energy metabolism dependency, but also effectively curbed their proliferation. Furthermore, VSi‐BP@HA has been demonstrated to potentiate CRC vulnerability to standard‐of‐care therapies, including chemotherapy (OHP) and immunotherapy (*α*PD‐1). On the one hand, VSi‐BP@HA inhibited drug resistance‐related protein synthesis and function by decreasing ATP levels in CRC cells, thereby reducing drug efflux and helping restore chemotherapy sensitivity in OHP‐resistant CRC. On the other hand, VSi‐BP@HA mediated immunogenic cell death of tumor cells and relieved the tumor immunosuppressive microenvironment, thereby potentiating the therapeutic efficacy of PD‐L1 immunotherapy.

Despite the notable efficacy of VSi‐BP@HA in disrupting tumor energy metabolism, there remain limitations that require attention and resolution in future research. A primary limitation lies in the introduction of inorganic silicon nanomaterials. Additionally, research on the therapeutic application of transition metal vanadium is still relatively scarce, necessitating further investigation into its specific roles in the body. The favorable biocompatibility observed in our study for VSi‐BP@HA was likely due to the limited dosage administered. It is necessary to conduct a more rigorous and comprehensive evaluation of VSi‐BP@HA toxicity. Nevertheless, overall, this study confirmed the promising potential of metabolic therapy in the treatment of CRC.

## 4 Experimental Section

### Regents

Cetyltrimethylammonium chloride (CTAC), Triethanolamine (TEA), bis‐2‐(5‐phenylacetamido‐1,2,4‐thiadiazol‐2‐yl)ethyl Sulfide (BPTES), Tetraethyl orthosilicate (TEOS), Methyl thiazolyl tetrazolium (MTT), 5,5'‐Dithiobis‐(2‐nitrobenzoic acid) (DTNB), Glutathione (reduced) (GSH), agarose (Low Melting Point) and 2‐[N‐(7‐Nitrobenz‐2‐oxa‐1,3‐diazol‐4 ‐yl)amino]‐2‐deoxy‐D‐glucose (2‐NBDG) and 2‐Deoxy‐D‐glucose (2‐DG) were purchased from Aladdin‐Reagent Co., Ltd. (China). Ethanol, Hydrochloric acid (HCl), and DMSO came from Shanghai Reagent Chemical Co. (China). Vanadium (III) chloride (VCl_3_), Oxaliplatin (OHP), and Triton X‐100 were obtained from Shanghai Macklin Biochemical Co., Ltd. (China). Hyaluronic acid (HA) came from Bloomage Freda Biopharm Co., Ltd. (China). ATP Assay Kit, RIPA lysis buffer, and BCA Protein Assay Kit were purchased from Beyotime Institute of Biotechnology (China). Annexin V‐FITC/PI cell apoptosis kit, Collagenase IV, Hyaluronidase, and DNase I were obtained from Yeasen (Shanghai, China). Pyruvate Kinase (PK) Activity Assay Kit, Lactate Dehydrogenase (LDH) Activity Assay Kit, Glucose‐6‐Phosphate Dehydrogenase (G6PD) Activity Assay Kit, Glutaminase (GLS) Activity Assay Kit, Mouse HMGB1 ELISA Kit and RBC lysis buffer came from Beijing Solarbio Science & Technology Co., Ltd. (China). Protease inhibitors and nonfat milk powder were purchased from GBCBIO Technologies Inc. (China). Prestained protein marker (MP102‐01) came from Vazyme Biotech Co., Ltd. (China). PAGE Gel Fast Preparation Kit (12.5%, PG113) was obtained from Shanghai Epizyme Biomedical Technology Co., Ltd. (China). Nitrocellulose (NC) membrane came from Pall China Co., Ltd. (China). Trypsin, Tris‐buffered Saline (TBS), tween 20, paraformaldehyde, bovine serum albumin, DAPI solution, and Phosphate‐buffered Saline (PBS) were obtained from Beijing Labgic Technology Co. Ltd. (China). Hexokinase 2 Polyclonal antibody (22029‐1‐AP), LDHA Polyclonal antibody (19987‐1‐AP), KGA/GAC Polyclonal antibody (12855‐1‐AP), G6PD Polyclonal antibody (25413‐1‐AP), GLUT1 Polyclonal antibody (21829‐1‐AP), PKM2‐specific Polyclonal antibody (15822‐1‐AP), PGP Polyclonal antibody (25081‐1‐AP), Beta Actin Monoclonal antibody (66009‐1‐Ig), HRP‐conjugated Affinipure Goat Anti‐Mouse IgG (SA00001‐1), HRP‐conjugated Affinipure Goat Anti‐Rabbit IgG (SA00001‐2), Calreticulin Polyclonal antibody (27298‐1‐AP), CoraLite488‐conjugated Goat Anti‐Rabbit lgG (SA00013‐2) and CoraLite594‐conjugated Goat Anti‐Rabbit lgG (SA00013‐4) were bought from Proteintech Group, Inc (China). Cleaved caspase‐3 Antibody (9661T), and Cytochrome c Monoclonal Antibody (11940T) were purchased from Cell Signaling Technology, Inc (America). FITC anti‐mouse CD11c Antibody (117306), APC anti‐mouse CD80 Antibody (104714), PE anti‐mouse CD86 Antibody (105008), FITC anti‐mouse CD3 Antibody (100204), APC anti‐mouse CD8 Antibody (100712), PE anti‐mouse CD4 Antibody (100408) and PE anti‐mouse IFN‐γ Antibody (163504) came from BioLegend, Inc. (America). Fixation / Permeabilization solution was bought from Becton, Dickinson and Company (America). TRIzol Reagent were purchased from Magen Biotechnology Co., Ltd. (China). The medium (DMEM/RPMI1640) came from Boster Biological Technology Co., Ltd. (China). Fetal bovine serum and Enhanced chemiluminescence (ECL) were purchased from Wuhan Huiyu Cheng Biotechnology Co., Ltd. (China).

### Preparation of sSiO_2_

The sSiO_2_ were synthesized based on previously reported experimental protocols.^[^ ^28^ ^]^ In detail, TEA (0.2 g) and CTAC (20 g) were dissolved in 200 mL deionized water with magnetic stirring at 80 °C for 20 min in an oil bath. Then TEOS (15 mL) was dropped into the solution and stirred for 4 h at 80 °C (350 rpm) to obtain the sSiO_2_. Next, the product was collected by centrifugation (11 000 rpm) for 40 min and washed with ethanol and water. Finally, to remove the residual CTAC, the product was re‐dispersed in 37 wt.% hydrochloric acid and ethanol solution (HCl: ethanol = 1:9) and stirred for 6 h at 60 °C (three times).

### Preparation of Vanadium‐Doped Hollow Mesoporous Silica Nanoparticles (VSi)

To obtain VSi, the as‐prepared sSiO_2_ (216 mg) and VCl_3_ (70.65 mg) were dispersed in 64.5 mL deionized water, then 12 mL NH_3_·H_2_O (30 wt.%) was added dropwise. The mixture was transferred to a polytetrafluoroethylene autoclave and placed in a heating furnace at 140 °C for 10 h. Finally, the green products were collected by centrifugation.

### Preparation of VSi‐BP@HA Nanoparticles

VSi (1 mL, 2 mg mL^−1^) was added dropwise into BPTES (267uL, 2 mM) solution with ultrasound for 10 min, and the precipitate was collected by centrifuged (12000 rpm, 15 min) and washed with deionized water to obtain VSi‐BP. Subsequently, VSi‐BP was re‐dispersed in 2 mL HA aqueous solution (10 mg mL^−1^) and sonicated for 30 min. Finally, VSi‐BP@HA was obtained by centrifugation (12000 rpm, 15 min) and washed with deionized water.

### Preparation of hSiO_2_ and Si‐BP@HA Nanoparticles

To obtain hSiO_2_, the as‐prepared sSiO_2_ (216 mg) were resuspended in 60 mL deionized water, then 12 mL NH_3_·H_2_O (30 wt.%) was added dropwise. The mixture was transferred to a polytetrafluoroethylene autoclave and placed in a heating furnace at 140 °C for 10 h. Finally, the white products were collected by centrifugation. Then, the collected hSiO_2_ (1 mL, 2 mg mL^−1^) was added dropwise into BPTES (267 µL, 2 mM) solution with ultrasound for 10 min, and the precipitate was collected by centrifugation (12000 rpm, 15 min) and washed with deionized water to obtain Si‐BP. Following that, Si‐BP was re‐dispersed in 2 mL HA aqueous solution (10 mg mL^−1^), sonicated for 30 min, and centrifuged (12000 rpm, 15 min) to obtain Si‐BP@HA.

### Degradation and V Release of VSi‐BP@HA In Vitro

2 mg of VSi‐BP@HA dispersed different buffer solutions (pH 5.5, 6.5, and 7.4) were put in dialysis bags (MWCO: 1000 Da) and immersed into 5 mL corresponding buffer at 37 °C in a horizontal laboratory shaker (37 °C, 100 rpm). At the given time interval, 2 mL of buffer solution was taken out and supplemented with 2 mL of corresponding fresh buffer solution. Finally, the concentrations of V in dialysate were detected by ICP‐MS. To evaluate degradation behavior, VSi‐BP@HA was immersed in different buffer solutions and centrifuged at predetermined time points to collect residual nanoparticles for analysis using UV–vis spectrometer and SEM.

### Cell Culture

Mouse colorectal tumor cells (CT26), mouse embryonic fibroblast cells (3T3) and mouse fibroblast cells (L929) were incubated in the medium (RPMI1640). Mouse colorectal tumor cells (MC38) and Human colorectal carcinoma cells (HCT116) were cultured in the medium (DMEM). OHP‐resistant HCT116 cells (HCT116/L‐OHP) were cultured in the medium (RPMI1640) containing 1.5 µg mL^−1^ OHP. All the mediums were supplemented with 10% fetal bovine serum (FBS), 100 units mL^−1^ penicillin, and 100 µg mL^−1^ streptomycin. All the cells were cultured at 37 °C under 5% CO_2_. An inverted microscope was used to monitor cell morphology and growth density on a regular basis. Unless otherwise specified, the cells were cultured in nutrient‐rich mediums (RPMI1640 containing 2000 mg L^−1^ glucose and 300 mg L^−1^ glutamine; DMEM containing 4500 mg L^−1^ glucose and 584 mg L^−1^ glutamine).

### MTT Cytotoxicity Assay In Vitro

Cell viability was assessed by MTT assay. Colorectal tumor cells (CT26, MC38, and HCT116/L‐OHP) were seeded in 96‐well plates (5 × 10^3^ cells per well) and cultured for 24 h. To determine the glutamine dependence of colorectal tumor cells (CT26 and MC38), cells were then cultured in glutamine‐deficient mediums with 2000 mg L^−1^ glucose and different concentrations of glutamine for 24 h. Similarly, to determine the glucose dependence of colorectal tumor cells, cells were then cultured in glucose‐deficient mediums with 300 mg L^−1^ glutamine and different concentrations of glucose for 24 h. To determine the cytotoxicity of BPTES and vanadium, different cells (CT26, MC38, 3T3, and L929) were cultured in mediums containing different concentrations of VCl_3_ (0–88 µM) or BPTES (0–19.8 µM) for 48 h. In addition, colorectal tumor cells (CT26 and MC38) were incubated in mediums containing different concentrations of Si@HA, VSi@HA, and VSi‐BP@HA for another 48 h to determine the cytotoxicity of nanoparticles. To determine the cytotoxicity of BPTES and VCl_3_ under nutrient‐deficient conditions, tumor cells were treated with different reagents (VCl_3_ 18 µM, BPTES 4 µM; VCl_3_ 40 µM, BPTES 2.6 µM) under different conditions (without glutamine or low‐concentration glucose (200 mg L^−1^)). Finally, MTT solution (20 µL, 5 mg mL^−1^) was added to each well and incubated for 4 h in the dark. The supernatant was replaced by DMSO (150 µL), and the optical density (OD) of each well was detected by a microplate reader at 570 nm. The relative cell viability was estimated by the following formula: cell viability (%) = (OD_sample_ – OD_blank_) / (OD_control_ – OD_blank_) × 100%, where OD_control_ and OD_blank_ represented the OD value of the well with untreated cells and no cells, respectively.

### Cell Proliferation Curve

To study the cell growth curve reconstruction under the action of BPTES or 2‐DG, colorectal tumor cells (CT26 and MC38) were seeded in 24‐well plates at a density of 800–3200 cells per well. Following that, cells were incubated with BPTES (4 µM) or 2‐DG (2 mM) for 1–5 days. Mediums containing BPTES or 2‐DG were refreshed at the mid‐time of the experiment. To study the cell growth curve under the combined action of VCl_3_ and BPTES, cells (CT26, MC38, 3T3, and L929) were cultured in 96‐well plates with a density of 3 × 10^3^ to 4.5 × 10^3^ cells per well. After 24 h, cells were treated with VCl_3_ (12 µM) and BPTES (1 µM) for 1–4 days. Similarly, cells were treated with VSi‐BP@HA (20 µg mL^−1^) for 1–4 days to study the cell growth curve.

### Analysis of Enzyme Activity

For the detection of enzyme activities of pyruvate kinase (PK), hexokinase (HK), lactate dehydrogenase (LDH), and glucose‐6‐phosphate dehydrogenase (G6PD), CT26 cells were plated in 6‐well plates (2 × 10^5^ cells per well) and cultured for 24 h. Then, the cells were incubated with VCl_3_ (18 µM), VCl_3_ (24 µM), BPTES (8 µM), VSi@HA (60 µg mL^−1^), and VSi‐BP@HA (60 µg mL^−1^) for another 24 h. Afterward, cells were harvested, and 1 mL of extraction was added to the obtained cell precipitates, followed by sonication on ice (200 W, 3 s, 30 cycles). Subsequently, the mixture was centrifuged at 8000 rcf for 10 min at 4 °C. The resulting supernatants were tested for the activity of each enzyme by the corresponding assay kits. For measurement of enzyme activity of glutaminase (GLS), CT26 cells were inoculated in 6‐well plates (2 × 10^5^ cells per well) for 24 h. Cells were then exposed to BPTES (8 µM), VSi@HA (60 µg mL^−1^), VSi‐BP@HA (60 µg mL^−1^) for an additional 24 h. Following this, the cells were collected, and 1 mL of extraction was introduced to the resulting cell precipitates, which were then subjected to sonication at 4 °C (300 W, 3 s, 18 cycles). Thereafter, the mixture was centrifuged at 12000 rcf for 15 min at 4 °C. The obtained supernatants were detected via a Glutaminase (GLS) activity assay kit.

### Evaluation of Oxygen Consumption

Partial pressure of oxygen (pO_2_) in cells was determined by the Oxygen Consumption Rate Assay Kit (MitoXpress‐Xtra HS Method). In brief, CT26 cells were seeded in 96‐well plates (5,000 cells per well) for 24 h, then cells were treated with GOX, PBS, BPTES (4 µM), VSi@HA (40 µg mL^−1^), or VSi‐BP@HA (40 µg mL^−1^) for 8 h before adding the oxygen consumption assay. Immediately after that, the medium was sealed with liquid paraffin wax to cut off the oxygen supply of cells according to the provider's protocol. After 2 h, pO_2_ in cells was assessed by detecting the fluorescence intensity. In addition, oxygen consumption in the medium was measured by a dissolved oxygen meter (JPSJ‐605F). In brief, CT26 cells were inoculated in 6‐well plates (1 × 10^6^ cells per well), and cultured for 24 h followed by replacing the medium with 3 mL of fresh medium containing BPTES (4 µM), VSi@HA (40 µg mL^−1^), or VSi‐BP@HA (40 µg mL^−1^). After 4 h of co‐culture, the medium was sealed with liquid paraffin wax, and the oxygen content in each well was monitored using an oxygen electrode.

### Detection of Glucose Uptake

The glucose uptake was evaluated using a fluorescent glucose analogue 2‐NBDG. In brief, CT26 cells were seeded in 6‐well plates (2 × 10^5^ cells per well) for 24 h. Then cells were incubated with VCl_3_ (18 µM), BPTES (6 µM), Si@HA (60 µg mL^−1^), Si‐BP@HA (60 µg mL^−1^), VSi@HA (60 µg mL^−1^), VSi‐BP@HA (60 µg mL^−1^) for another 24 h. Afterward, the medium was removed and cells were stained with 2‐NBDG (100 µM, 1 mL) for 30 min, followed by washing three times with PBS. At last, cells were harvested and analyzed by flow cytometry.

### Detection of GSH

According to the method published in the previous literature, the GSH was detected using Ellman's reagents.^[^ ^62^ ^]^ In detail, CT26 cells were plated in 6‐well plates (2 × 10^5^ cells per well) and cultured for 24 h. Afterward, different samples (Si@HA, Si‐BP@HA, VSi@HA, VSi‐BP@HA, 30 µg mL^−1^) were incubated with CT26 cells for 24 h. Then all cells were collected and washed three times with PBS, followed by adding 200 µL of Triton‐X‐100 lysate (0.4%) to lyse the cells. After centrifugation (6000 rpm, 5 min) of the lysates, 50 µL of supernatant was added to DTNB solution (200 µL, 0.5 mM). Finally, the microplate reader was used to measure the absorbance at 405 nm.

### Tumor Spheroid Assay

According to methods of the published literature with some modification,^[^ ^63^ ^]^ liquid agarose (1%, low melting point) was added to the 96‐well plates (50 µL per well). After the agarose solidified, CT26 cells were seeded in wells (600 cells per well) and stood for 5 days. Subsequently, the formed tumor spheroids were incubated with BPTES (8 µM), VCl_3_ (36 µM), BPTES+VCl_3_, Si@HA (30 µg mL^−1^), VSi@HA (30 µg mL^−1^), VSi‐BP@HA (30 µg mL^−1^), and mediums were refreshed at specific incubation time points (24, 72, 120, 168, and 216 h), respectively. Tumor spheroids were photographed with a microscope imaging system every other day since the start of treatment. Tumor spheroid volumes were calculated by the formula: V = 0.5 × W^2^ × L (W and L represent the shortest diameter and longest diameter respectively).

### Analysis of the Release of DAMPs In Vitro

The content of HMGB1 released in medium was detected by ELISA. In detail, MC38 cells were plated in 6‐well plates (3 × 10^5^ cells per well) for 24 h. Then cells were incubated with Si‐BP@HA, VSi@HA, VSi‐BP@HA (80 µg mL^−1^) for another 12 h. After that, the medium was aspirated and centrifuged (12 000 rpm, 5 min), the obtained supernatant was measured by Mouse HMGB1 ELISA kit. The calreticulin (CRT) exposed on the surface of MC38 cells was analyzed by laser scanning confocal microscopy and flow cytometry. Specifically, MC38 cells were inoculated in a confocal dish (2 × 10^5^ cells per well) for 24 h. Subsequently, the cells were treated with Si‐BP@HA, VSi@HA, VSi‐BP@HA (80 µg mL^−1^) for another 12 h. The cells were then washed with PBS and fixed with paraformaldehyde (4%), followed by incubation with blocking buffer (5% bovine serum albumin) for 1 h. Afterward, the cells were incubated with calreticulin Polyclonal antibody overnight at 4 °C. Then, the cells were rinsed with PBS carefully, stained with CoraLite488‐conjugated Goat Anti‐Rabbit lgG for 1 h and incubated with DAPI staining solution for 10 min before observation. Similarly, the pre‐treated cells were stained with CoraLite594‐conjugated Goat Anti‐Rabbit lgG and collected for flow cytometry analysis.

### Evaluation of DC Maturation In Vitro

The maturation of DC cells was detected by flow cytometry. MC38 cells were inoculated in 6‐well plates (2 × 10^5^ cells per well) for 24 h. Then cells were incubated with Si‐BP@HA, VSi@HA, VSi‐BP@HA (80 µg mL^−1^) for another 12 h. Afterward, the medium was co‐incubated with DC cells (3 × 10^5^ cells) extracted from the bone marrow of healthy C57BL/6 mice for 24 h. All the cells were collected and stained with FITC anti‐mouse CD11c Antibody, APC anti‐mouse CD80 Antibody and PE anti‐mouse CD86 Antibody at 4 °C for 30 min. Subsequently, the cells were washed with PBS and detected by flow cytometry.

### Animals

Female BALB/c mice (5 to 6 weeks) were purchased from Shulaibao (Wuhan) Biotechnology Co., Ltd. Female BALB/c nude mice (4 to 5 weeks, Immunodeficient mice) and female C57BL/6 mice (5 to 6 weeks) were purchased from Ltd. Changsheng (Liaoning) biotechnology Co., Ltd. All animal researches were performed following the guidelines approved by the Institution Animal Care and Use Committee (IACUC) at Tongji Medical College, Huazhong University of Science and Technology, Wuhan, China (IACUC Number: 3680). CT26 tumor‐bearing mice were established by subcutaneous injection of CT26 cells (4 × 10^5^ cells per mouse) into the right flank of BALB/c mice. To construct a drug‐resistant HCT116/L‐OHP tumor‐bearing mouse model, HCT116/L‐OHP cells (1 × 10^7^ cells per mouse) were subcutaneously inoculated in the right flank of BALB/c nude mice. To establish an MC38 tumor‐bearing mouse model, MC38 cells (4 × 10^5^ cells per mouse) were subcutaneously injected into the right flank of C57BL/6 mice. All mice were maintained in Tong Ji Medical College's specific pathogen‐free (SPF) animal room (Wuhan, China).

### Distribution of VSi‐BP@HA In Vivo

To visualize the distribution of nanoparticles in mice, a Cyanine 5.5 fluorescent probe was used. Typically, VSi‐BP (100 mg) and Cyanine 5.5 (20 mg) were dispersed in 4 mL of ethanol with magnetic stirring for 4 h. The Cyanine 5.5‐labeled VSi‐BP nanoparticles were collected by centrifugation and then re‐dispersed in HA aqueous solution and magnetically stirred for 2 h. Finally, the Cyanine 5.5‐labeled VSi‐BP@HA (VSi‐BP@HA^cy5.5^) was collected by centrifugation (13 000 rpm, 25 min) and washed twice with deionized water. When the tumor volume reached ≈200 mm^3^, all CT26 tumor‐bearing mice were intravenously injected with VSi‐BP@HA^Cy5.5^. The fluorescence images were obtained at various times using the IVIS Imaging Spectrum System. Finally, mice were sacrificed and the main organs (heart, liver, lung, spleen, and kidney) and tumors were collected for ex vivo imaging at 24 h and 48 h post‐injection.

### In Vivo Antitumor Effect

To evaluate the therapeutic effect of VSi‐BP@HA in vivo, CT26 tumor‐bearing mice were established. When tumor volumes reached ≈25 mm^3^, the mice were randomly grouped (n = 6) and injected with PBS, BPTES (5 mg kg^−1^, *i.p*.), Si‐BP@HA (10 mg kg^−1^, *i.v*.), VSi@HA (10 mg kg^−1^, *i.v*.), VSi‐BP@HA (10 mg kg^−1^, *i.v*.) every 3 days for four times. The weight and tumor volume of the mice were recorded daily. The tumor volume (V) was calculated by the formula: V = L × W^2^ × 0.5. On day 14, all mice were sacrificed, tumors and major organs were divested for further analysis. For biosafety evaluation, major organs (heart, liver, spleen, kidney, lung) were sectioned into slices for H&E. For apoptosis and proliferation evaluation, immune‐fluorescence analysis of tumor slices was applied to estimate the expression of Cleaved caspase‐3, Ki‐67. To study the effect of VSi‐BP@HA sensitizing chemotherapy, drug‐resistant HCT116/L‐OHP tumor‐bearing mice were constructed. When tumor volumes reached ≈80 mm^3^, all the mice were randomly divided into three groups (n = 7). Mice were treated with PBS, oxaliplatin (10 mg kg^−1^, *i.p*.), VSi‐BP@HA (10 mg kg^−1^, *i.v*.), or VSi‐BP@HA+OHP every 3 days four times, respectively. The body weight and tumor volume were measured and calculated every day. All mice were sacrificed at the end of the experiment, the tumors were isolated for H&E staining, TUNEL immunofluorescence staining and P‐gp immunohistochemical analysis. To explore the effect of VSi‐BP@HA with the immune checkpoint inhibitor *α*PD‐1, MC38 tumor‐bearing mice were set up. As the volume of the tumors approached ≈45 mm^3^, all the mice were randomly segmented and injected with PBS, Si‐BP@HA (10 mg kg^−1^, *i.v*.), VSi@HA (10 mg kg^−1^, *i.v*.), VSi‐BP@HA (10 mg kg^−1^, *i.v*.), *α*PD‐1 (30 µg per mouse, *i.p*.), *α*PD‐1+VSi‐BP@HA every 3 days for 4 times. The body weight of the mice was measured daily and the tumor volume was calculated every day. All mice were executed on day 14 and the tumors were excised. Then, the tumors and spleens were isolated for flow cytometry.

### Statistical Analysis

Statistical analyses were conducted by the Graph‐Pad Prism 8.4. All data were displayed in the form of mean ± SD from at least three independent experiments. A two‐tailed, unpaired student's t‐test was employed to determine the statistical difference between the two groups. For comparisons of three or more groups, statistical significance was analyzed by one‐way ANOVA followed by Bonferroni's test. Significant differences were indicated by *p < 0.05, **p < 0.01, ***p < 0.001, ns, not significant.

## Conflict of Interest

The authors declare no conflict of interest.

## Supporting information



Supporting Information

## Data Availability

The data that support the findings of this study are available from the corresponding author upon reasonable request.
