# Supplementary material for: Targeting Metabolic Adaptation of Colorectal Cancer with Vanadium‐Doped Nanosystem to Enhance Chemotherapy and Immunotherapy
Source: Adv Sci (Weinh). 2024 Dec 30;12(7):2409329. doi: 10.1002/advs.202409329 (PMC11831457; doi:10.1002/advs.202409329)
Supplement: Supplementary file 1 — Supporting Information [file ADVS-12-2409329-s001.pdf]

ADVANCED SCIENCE
Open Access

# Supporting Information

for *Adv. Sci.*, DOI 10.1002/advs.202409329

Targeting Metabolic Adaptation of Colorectal Cancer with Vanadium-Doped Nanosystem to Enhance Chemotherapy and Immunotherapy

*Qian Cheng, Yuzhe Chen, Danyi Zou, Qilin Li, Xiaolei Shi, Qushuhua Qin, Miaodeng Liu, Lin Wang* and Zheng Wang**

# Supporting Information

**Targeting Metabolic Adaptation of Colorectal Cancer with Vanadium-Doped Nanosystem to Enhance Chemotherapy and Immunotherapy**

*Qian Cheng*[1, 2, 4, 5§], *Yuzhe Chen*[1, 2, 4, 5§], *Danyi Zou*[1, 2, 4], *Qilin Li*[1, 2, 4, 5], *Xiaolei Shi*[1, 2, 4, 5], *Qushuhua Qin*[1, 3, 4, 5], *Miaodeng Liu*[1, 2, 4, 5], *Lin Wang*[1, 2, 4, 5]*, *and Zheng Wang*[1, 3, 4, 5]*

[1]Research Center for Tissue Engineering and Regenerative Medicine, Union Hospital, Tongji Medical College, Huazhong University of Science and Technology, Wuhan, 430022, China.
[2]Department of Clinical Laboratory, Union Hospital, Tongji Medical College, Huazhong University of Science and Technology, Wuhan, 430022, China.
[3]Department of Gastrointestinal Surgery, Union Hospital, Tongji Medical College, Huazhong University of Science and Technology, Wuhan, 430022, China.
[4]Hubei Key Laboratory of Regenerative Medicine and Multi-disciplinary Translational Research, Wuhan, 430022, China.
[5]Hubei Provincial Engineering Research Center of Clinical Laboratory and Active Health Smart Equipment, Wuhan 430022, China

*Corresponding author. Email: lin_wang@hust.edu.cn (L.Wang.), zhengwang@hust.edu.cn (Z.Wang.)
§These authors contributed equally: Qian Cheng, Yuzhe Chen

**Supplementary Experimental Sections**

*Experimental apparatus*: The morphology of nanoparticles was characterized by scanning electron microscopy (SEM, Zeiss SIGMA) and transmission electron microscopy (TEM, JEM-2100 microscope). For element mapping images, high-angle annular dark field scanning transmission electron microscopy (HAADF STEM) was used. An X-ray photoelectron spectrometer (XPS, ESCALAB 250Xi XPS, Thermo Fisher Scientific) was used to characterize the chemical states of elements in nanoparticles. UV−vis absorption spectra of all samples were detected by a UV-vis spectrophotometer (Lambda Bio40 UV/Vis, Perkin-Elmer). Nano-ZS ZEN3600 (nano-ZS90) was applied to measure the hydrodynamic size and zeta potential of all nanoparticles. The V content in VSi was determined by inductively coupled plasma-mass spectrometry (ICP-MS, Agilent 7800). Flow cytometry was performed using a Flow cytometer (LSRFortessa X20 Flow Cytometry System, BD Bioscience). Distribution of nanoparticles *In Vivo via In Vivo* Imaging System (IVIS Imaging Spectrum System, PerkinElmer).

*Dispersibility and stability assay*: The hydrodynamic size and polydispersity Index of VSi-BP@HA dispersed in PBS at different time points (0 h, 2 h, 6 h, 12 h, 24 h, 48 h) were monitored. VSi-BP@HA were immersed in water and PBS and photographed at different times to observe their dispersibility.

*Hemolysis assay*: The whole blood from BALB/c mice was centrifuged (3000 rpm, 5 min) to collect red blood cells (RBCs) and washed with PBS until the supernatant was colorless. Subsequently, RBCs were dispersed into PBS buffers containing different concentrations of VSi-BP@HA (0-250 mg $L^{-1}$). RBCs incubated with Triton X-100 (0.4 %) and PBS were set as control groups. After 1 h of co-incubation, all samples were centrifuged and photographed, and the absorbance of supernatants at 540 nm was measured.

*Western blotting*: Total protein from pre-treated cells in 6-well plates was extracted with RIPA (containing protease inhibitors) lysis buffer at 4 °C, followed by sonication (3 s, 5 cycles) and centrifugation (12,000 rpm, 15 min) at 4 °C. The BCA detection assay was applied to measure the supernatant after eliminating any insoluble substances. Immediately, the protein was denatured at 100 °C for 10 min, after which identical amounts of protein (40 μg) and prestained protein marker (3 μL) were loaded in a 12% sodium dodecyl sulfate-polyacrylamide gel (SDS-PAGE) and then transferred to a NC membrane. Later, the membranes were washed with Tris-buffered Saline (TBS) and Tween 20 (TBST) buffer for 3 times, followed by 1 hour of blocking

in blocking buffer (5% nonfat milk or 5% bovine serum albumin), and washed with TBST buffer for 3 times again. Afterward, the membranes were incised into strips and incubated with primary antibody at 4 °C overnight. Finally, all the strips were rinsed with TBST buffer 3 times, cultured with the corresponding secondary antibody at room temperature for 1 h, and washed with TBST buffer 3 times. Enhanced chemiluminescence (ECL) was used for protein visualization. The Image J software was utilized to measure the grey value of the target bands, and the relative expressions were computed as the gray value ratio of the target protein to loading control. To access the expressions of hexokinase 2 (HK2), lactate dehydrogenase A (LDHA), glutaminase 1 (GLS1), glucose-6-phosphate dehydrogenase (G6PD), Glucose transporter type 1 (GLUT1), and pyruvate kinase isozyme type M2 (PKM2) in colorectal tumor cells and normal cells, CT26, MC38, L929, and 3T3 cells were cultured in 6-well plates ($2\times10^5$ cells per well) for 24 h, respectively. Subsequently, the cells were gathered and examined through the aforementioned Western blotting assay, employing the following primary antibodies: Hexokinase 2 Polyclonal antibody, LDHA Polyclonal antibody, KGA/GAC Polyclonal antibody, G6PD Polyclonal antibody, GLUT1 Polyclonal antibody, and PKM2-specific Polyclonal antibody. Similarly, to evaluate the expressions of HK2, LDHA, GLS, G6PD, GLUT1, and PKM2 in tumor cells under nutrient-deficient conditions, CT26 were plated in 6-well plates ($2\times10^5$ cells per well) for 24h, followed by refresh the medium with various concentration of glucose (889-4500 mg $L^{-1}$) or glutamine (26.3-450 mg $L^{-1}$) for another 24h. Then, the cells were collected and analyzed as mentioned above. To analyze the expression of caspase 3 and cytochrome c in tumor cells treated with different samples, CT26 cells were inoculated in 6-well plates ($2\times10^5$ cells per well) for 24h. After the medium was removed, BPTES (8 μM), $VCl_3$ (18 μM), BPTES + $VCl_3$, Si@HA (40 μg $mL^{-1}$), Si-BP@HA (40 μg $mL^{-1}$), VSi@HA (40 μg $mL^{-1}$), VSi-BP@HA (40 μg $mL^{-1}$) were incubated with cells for 24h. Primary antibodies were used as follows: Cleaved Caspase-3 Antibody, Cytochrome c Monoclonal Antibody. To verify the expression of P-glycoprotein in tumor cells and drug-resistant tumor cells, HCT116 cells and HCT116/L-OHP cells were cultured in 6-well plates ($3\times10^5$ cells per well) for 24h. Afterward, the cells were harvested and analyzed by the primary antibody PGP Polyclonal antibody. To determine the expression of P-glycoprotein in HCT116/L-OHP cells treated with different nanoparticles, HCT116/L-OHP cells were inoculated in 6-well plates ($3\times10^5$ cells per well). After 24h, the mediums were replaced with RPMI1640 containing BPTES (8 μM), VSi@HA (40 μg $mL^{-1}$), VSi-BP@HA (40 μg $mL^{-1}$) for 24h. then, the cells were collected and examined using the primary antibody PGP Polyclonal antibody.

*Detection of ATP*: Colorectal tumor cells (CT26, MC38, HCT116/L-OHP) were plated in 6-well plates ($2\times10^5$ cells per well) and cultured for 24h. Then they were cultured in different nutrient environments (nutrient-rich, only containing glutamine, and only containing glucose) for 12 h or 24 h to determine the dependence of ATP production on glutamine and glucose. Subsequently, the cells were collected and counted, lysed on ice, and then centrifuged (12000 g, 5 min) at 4°C. Eventually, the supernatants were measured by an ATP assay kit. In parallel, to determine the effect of nanoparticle treatment on ATP production by tumor cells, colorectal tumor cells (CT26, HCT116/L-OHP) were treated with different nanoparticles (Si@HA, Si-BP@HA, VSi@HA, VSi-BP@HA, 40 μg $mL^{-1}$).

*Fenton-like reaction of VSi-BP@HA:* The VSi-BP@HA (100 μg $mL^{-1}$), TMB (1 mM) and different concentrations of $H_2O_2$ (0, 20, 40, and 80 mM) were incubated in pH 6.5 buffer solutions for various time (0-20 min). The VSi-BP@HA (100 μg $mL^{-1}$), TMB (1 mM) and $H_2O_2$ (80 mM) were dispersed in different buffer solutions (pH 5.0, pH 6.5, pH 7.4) for various time (0-60 min). At the end time, a microplate reader was used to measure the absorbance at 652 nm. UV-vis spectrophotometer was used to detect the spectra of TMB in different solutions.

*Measurement of energy metabolites in CT26 tumor cells*: CT26 cells were seeded in 6-well plates ($2\times10^5$ cells per well) for 24h. Then, the supernatant was discarded, followed by washing with PBS twice. Afterward, the cells were exposed to $VCl_3$ (40 μM) and VSi@HA (80 μg $mL^{-1}$) for 24 h, and cells incubated with only complete DMEM were used as control. After treatment, the cells were washed with PBS and harvested. The cell counter was applied to take $1\times10^7$ cells and the obtained cells were frozen in liquid nitrogen and temporarily stored at -80 °C. For metabolite extraction, the samples were taken from -80 °C and added to 1 ml of methanol/acetonitrile/$H_2O$ solution (2:2:1, v/v/v) and 10 μL SUCCINIC ACID-D6 internal standard. The resulting samples were vortexed for 60 s, sonicated at low temperature for 30 min (twice), and the protein was then precipitated at -20 °C for 1 h. Subsequently, the mixture was centrifuged (14000 rcf) at 4 °C for 20 minutes, the obtained supernatant was freeze-dried and preserved at -80 °C. Separation of the samples was achieved using ultra-high-performance liquid chromatography (Agilent 1290 Infinity LC), and analysis was conducted by a mass spectrometer (5500 QTRAP, AB SCIEX) in negative ion mode.

*Apoptosis Evaluation by Flow Cytometric Analysis*: CT26 cells were inoculated on 6-well plates ($2\times10^5$ cells per well). After being cultured for 24h, cells were treated with $VCl_3$ (36 μM), BPTES (4 μM), Si-BP@HA (60 μg $mL^{-1}$), VSi@HA (60 μg $mL^{-1}$), VSi-BP@HA (60 μg $mL^{-1}$) for 24 h. Cells treated only with PBS or Si@HA (60 μg $mL^{-1}$) were used as controls. Then, all cells were carefully washed with PBS and digested with trypsin (without EDTA). Finally, all cells were collected, stained with annexin V-FITC and PI, and analyzed by flow cytometry.

*Detection of intracellular Reactive Oxygen Species (ROS)*: Intracellular ROS levels were detected using flow cytometry, and DCFH-DA was used as a ROS probe. CT26 cells were cultured in 6-well plates ($2\times10^5$ cells per well) for 24h. Then cells were exposed to Si@HA, Si-BP@HA, VSi@HA, VSi-BP@HA (40 μg $mL^{-1}$) for another 24h. Afterward, the medium was removed and all cells were stained with DCFH-DA for 20 min. Eventually, cells were collected and analyzed by flow cytometry.

*Biocompatibility of Nanoparticles In Vivo*: In brief, healthy BALB/c mice were randomly divided into three groups (n = 3), followed by different treatments (VSi-BP@HA (10 mg $kg^{-1}$) by intravenous injection, free BPTES (5 mg $kg^{-1}$) by intraperitoneal injection and oral gavage). After 3 days, blood samples were collected *via* cardiac puncture for blood biochemistry and blood routine assays.

*Distribution of Nanoparticles and Pharmacokinetics In Vivo:* CT26 tumor-bearing mice were used to evaluate the distribution of VSi-BP@HA. When tumor volumes reached around 200 $mm^3$, the mice were injected intravenously with VSi-BP@HA (administer a dosage with a Si content of 5 mg $kg^{-1}$), at different time points (1 h, 4 h, 8 h, 12 h, 24 h, 48 h) after the injection the mice were sacrificed and major organs, blood, tumors were collected. Then, all samples were dissolved in aqua regia at 90 °C, and the amount of Si was measured using ICP-MS.

*Method for RNA-Seq of CT26 tumors*: CT26 tumor-bearing mice were constructed for RNA-Seq. When tumor volumes reached about 80 $mm^3$, the mice were randomly grouped (n = 3) and injected with PBS or VSi-BP@HA (10 mg $kg^{-1}$, *Intratumoral*) every day for 4 times. Next, the mice were executed and the tumors were isolated, cleansed of blood stains and preserved at -80 °C temporarily. The obtained tumors were treated with TRIzol® Reagent for total RNA extraction. The Nanodrop ND-2000 (Thermo Scientific, USA) and Agilent Bioanalyzer 4150 (Agilent Technologies, CA, USA) were used to check the A260/A280 absorbance ratio and

RIN of the RNA samples to ensure all the samples were qualified. Then, the preparation of the paired-end libraries was carried out using an ABclonal mRNA-seq Lib Prep Kit (ABclonal, China). The mRNA was purified from 1 μg of total RNA using oligo (dT) magnetic beads, followed by mRNA fragmentation in ABclonal First Strand Synthesis Reaction Buffer and subsequent PCR amplification. Purification of the PCR product was performed (AMPure XP system), followed by an assessment of the library's quality on an Agilent Bioanalyzer 4150. Ultimately, the library preparations were sequenced on an MGISEQ-T7 and 150 bp paired-end reads were generated.

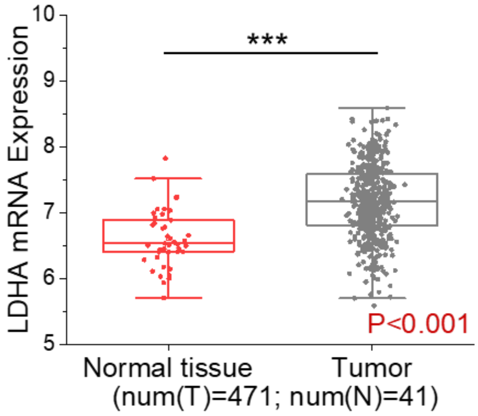


**Figure S1.** Relative levels of LDHA mRNA from microarray analysis (normalized log2 ratios) of primary tumor samples relative to adjacent normal tissue from cancer patients (the Cancer Genome Atlas (TCGA) database) are shown (https://genome-cancer.ucsc.edu). Data were statistically calculated by the two-tailed unpaired student's t-test. *$p$ < 0.05, **$p$ < 0.01, ***$p$ < 0.001.

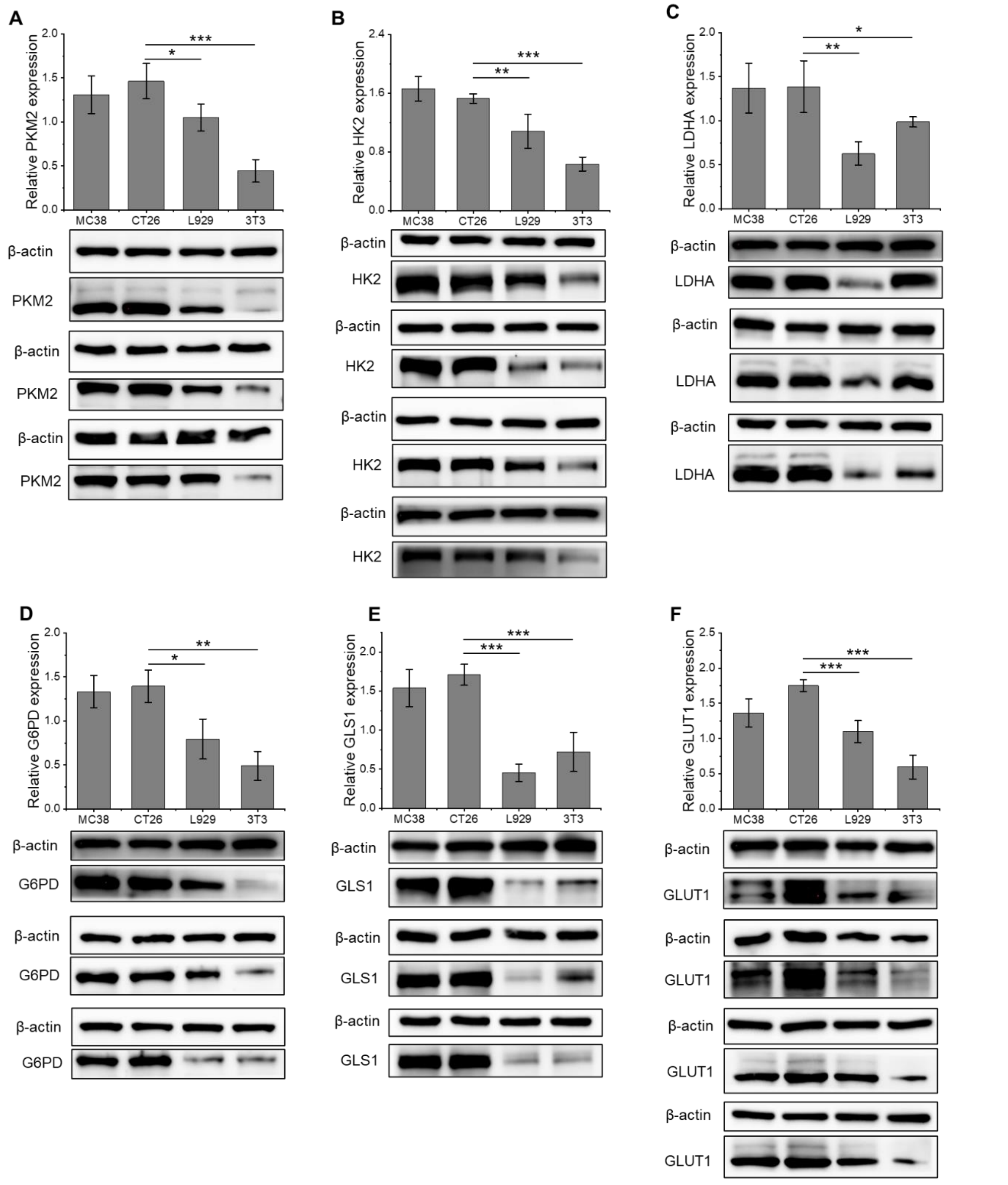


**Figure S2.** The expression of glucose metabolism-related enzymes and GLS1 in different cells. Semi-quantitative plots of the expression of A) PKM2 (n = 3), B) HK2 (n = 4), C) LDHA (n = 3), D) G6PD (n = 3), E) GLS1 (n = 3), and F) GLUT1 (n = 4) in CRC cells and normal cells. Data were represented as mean values ± SD. and statistically calculated by one-way ANOVA. *$p < 0.05$, **$p < 0.01$, ***$p < 0.001$.

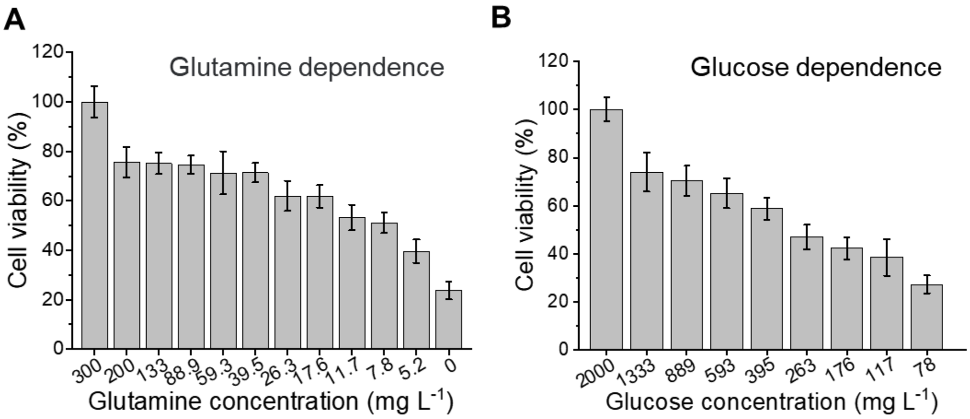


**Figure S3.** The glucose and glutamine dependencies of MC38 cells. A) Cell viability of MC38 cells grown with 2000 mg $L^{-1}$ glucose and different concentrations of glutamine (0 - 300 mg $L^{-1}$, *n* = 4), and B) cell viability of MC38 cells grown with 300 mg $L^{-1}$ glutamine and different concentrations of glucose (78-2000 mg $L^{-1}$, *n* = 4). Data were represented as mean values ± SD.

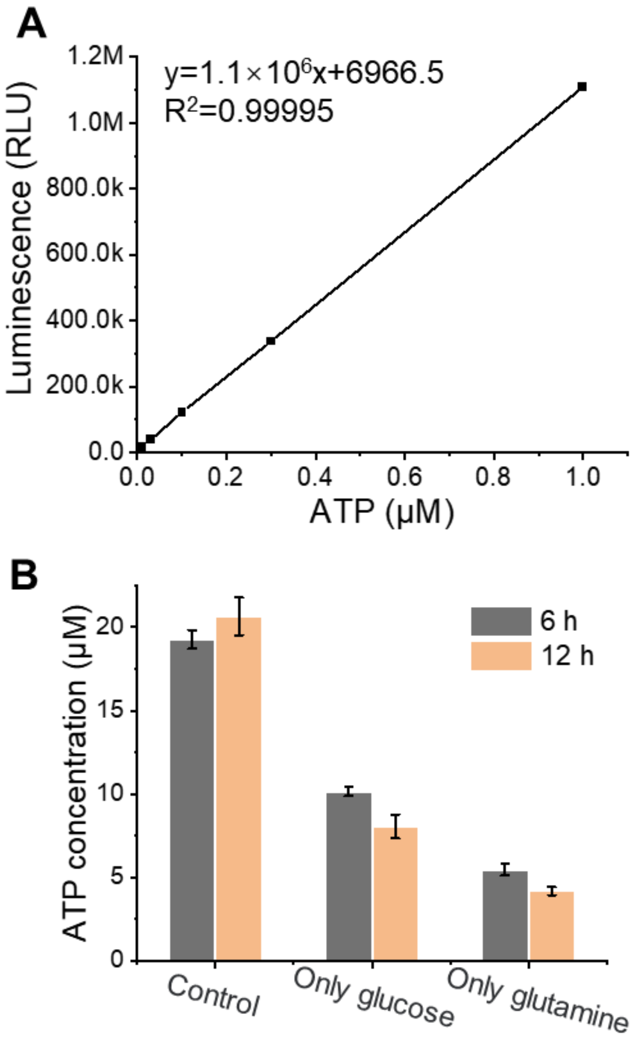


**Figure S4.** The ATP production in MC38 cells affected by different nutritional conditions. A) Standard curve for ATP detection; B) The ATP production in MC38 cells grown with complete DMEM, glutamine-only DMEM, and glucose-only DMEM (*n* = 3). Data were represented as mean values ± SD.

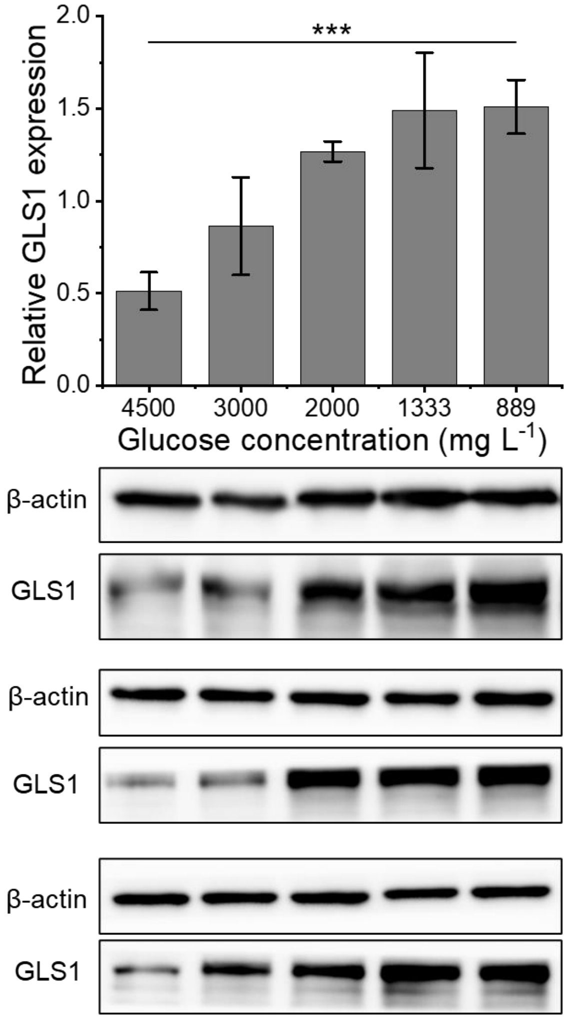


**Figure S5.** GLS1 expression at different glucose concentrations (889 - 4500 mg $L^{-1}$) (n = 3). Data were represented as mean values ± SD. and statistically calculated by the two-tailed unpaired student's t-test. **p* < 0.05, ***p* < 0.01, ****p* < 0.001.

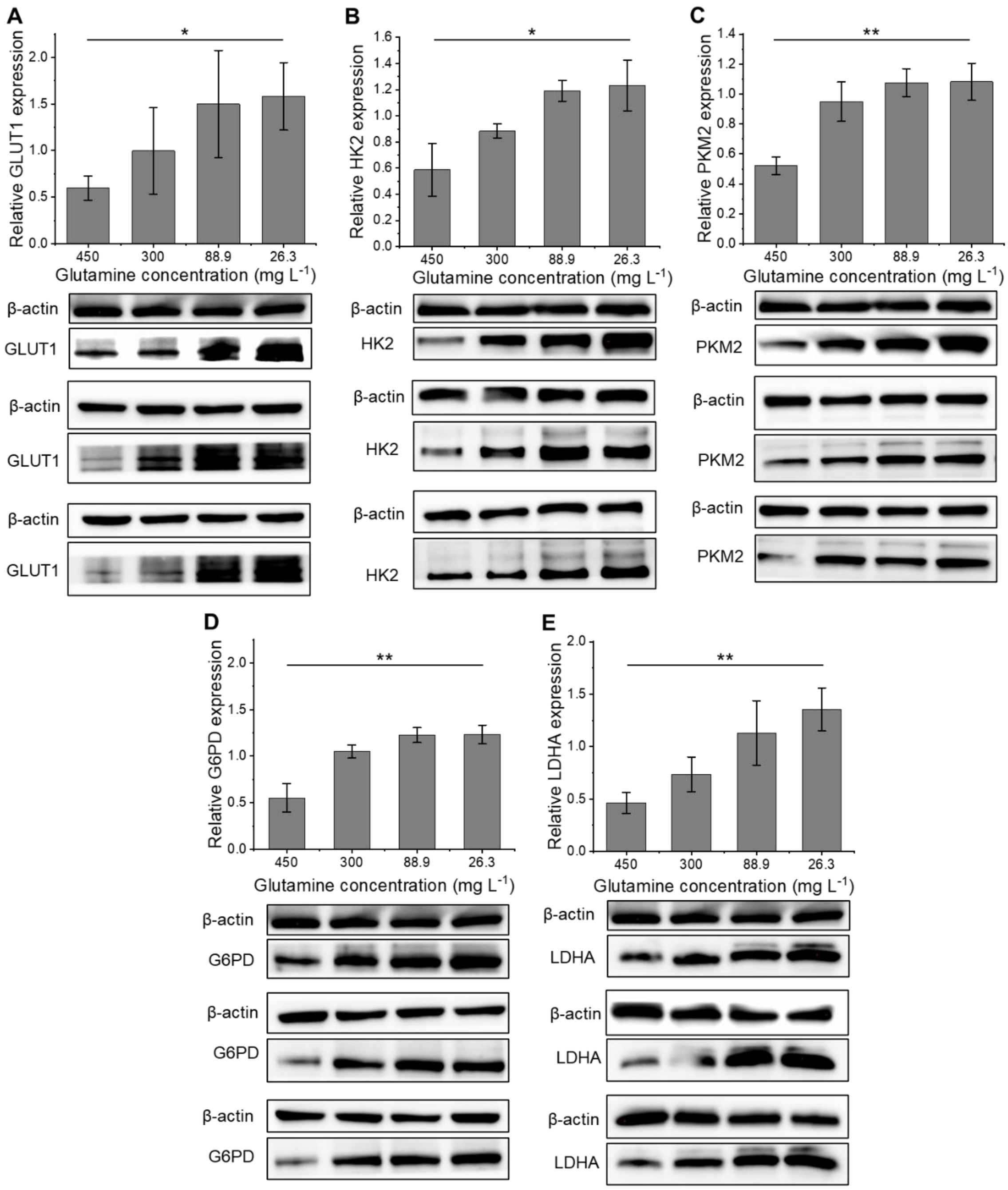


**Figure S6.** The glucose metabolism-related enzymes' expression at different glutamine concentrations. The expression of A) GLUT1, B) HK2, C) PKM2, D) G6PD, and E) LDHA at various glutamine concentrations (26.3 - 450 mg $L^{-1}$) (n = 3). Data were represented as mean values ± SD. and statistically calculated by the two-tailed unpaired student's t-test. *$p < 0.05$, **$p < 0.01$, ***$p < 0.001$.

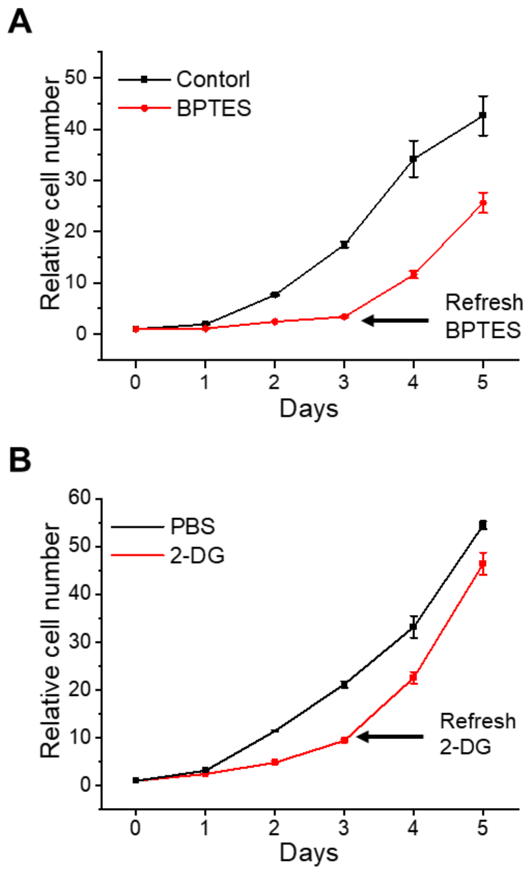


**Figure S7.** Relative proliferation of MC38 cells treated with BPTES or 2-DG. A) Relative proliferation of MC38 cell line treated with BPTES (4 μM) or untreated. As indicated BPTES was refreshed at the mid-point of the experiment (*n* = 4). B) Relative proliferation of MC38 cell line treated with 2-DG (2 mM) or untreated. As indicated BPTES was refreshed at the mid-point of the experiment (*n* = 4). Data were represented as mean values ± SD.

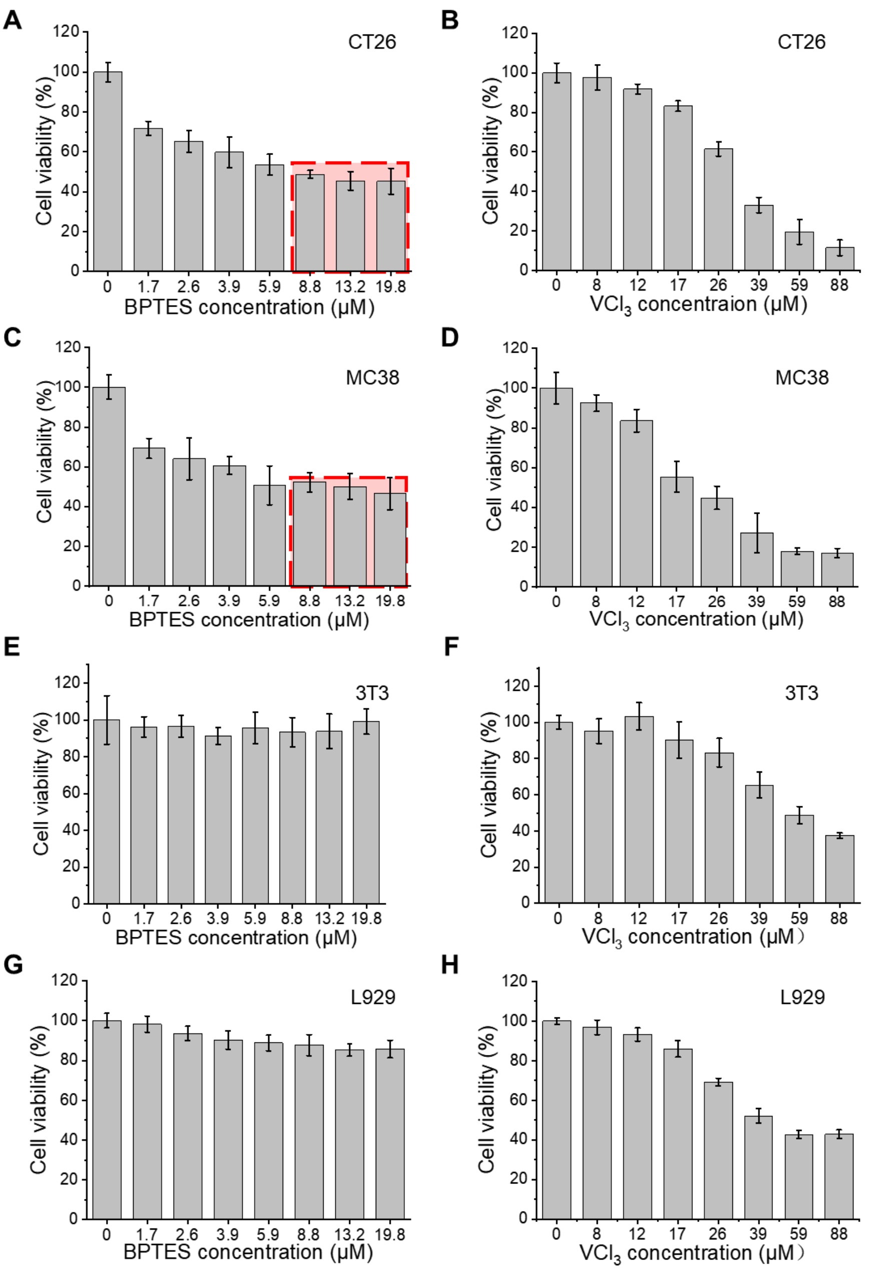


**Figure S8.** The cytotoxicity of $VCl_3$ and BPTES to different cells. A) CT26 tumor cells, C) MC38 tumor cells, E) 3T3 cells, G) L929 cells treated with different concentrations of BPTES. B) CT26 tumor cells, D) MC38 tumor cells, F) 3T3 cells, H) L929 cells treated with different concentrations of BPTES. Co-cultured for 48h ($n$ = 8). Data were represented as mean values ± SD.

A

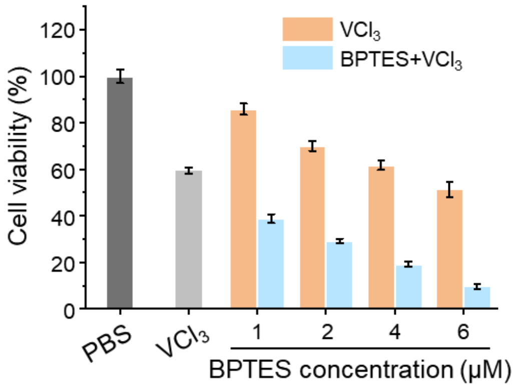


B

| BPTES concentration | $VCl_3$ concentration | Combination index |
|---|---|---|
| 2 μM | 8 μM | 1.66 |
| 2 μM | 12 μM | 1.77 |
| 2 μM | 17 μM | 1.78 |
| 2 μM | 26 μM | 1.38 |
| 1 μM | 17 μM | 1.25 |
| 2 μM | 17 μM | 1.21 |
| 4 μM | 17 μM | 1.28 |
| 6 μM | 17 μM | 1.30 |

c

**Figure S9.** Synergistic effect of BPTES and $VCl_3$ on killing CT26 cells. A) Cells were treated with 17 μM $VCl_3$ and different concentrations of BPTES ($n$ = 3). B) Jin's formula was employed to calculate the synergistic effect of the BPTES and $VCl_3$ combination treatment. The formula is: $Q = E_{a+b} / (E_a + E_b - E_a \times E_b)$, in which Q represents the combination index; $E_{a+b}$ represents the cell proliferative inhibition rate of the combined drug; $E_a$ and $E_b$ represent the cell proliferative inhibition rates of BPTES and $VCl_3$, respectively. Q>1.15, 0.85<Q<1.15, and Q<0.85 indicate synergy, additive effect, and antagonism, respectively.

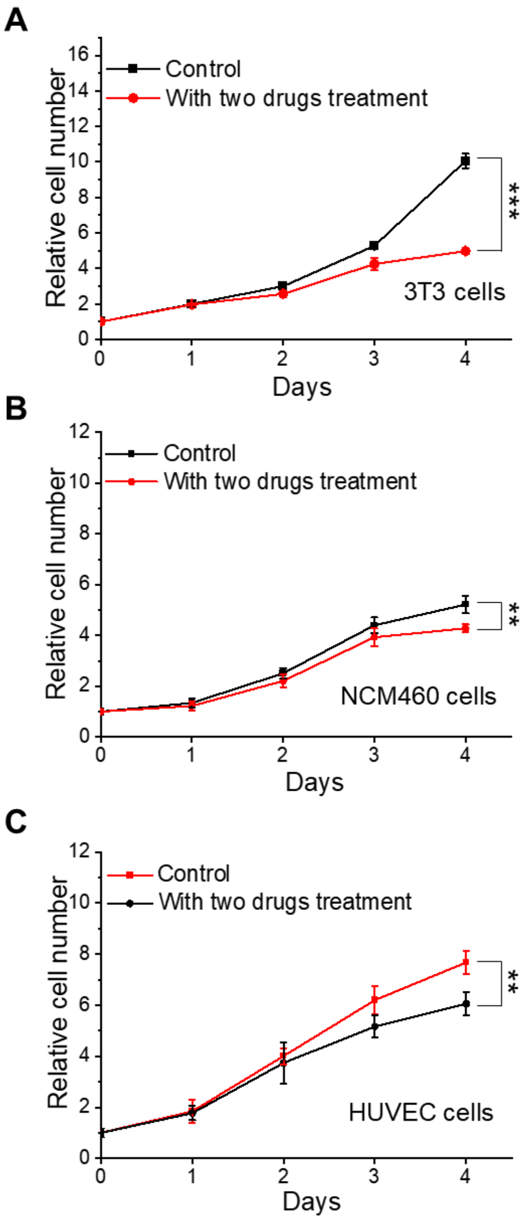


**Figure S10.** Effect of combination therapy (BPTES: 1 μM, $VCl_3$: 12 μM) on the proliferation of A) 3T3 cells, B) NCM460 cells, and C) HUVEC cells. $n = 8$. Data were represented as mean values ± SD. Data were statistically calculated by the two-tailed unpaired student's t-test. $*p < 0.05$, $**p < 0.01$, $***p < 0.001$.

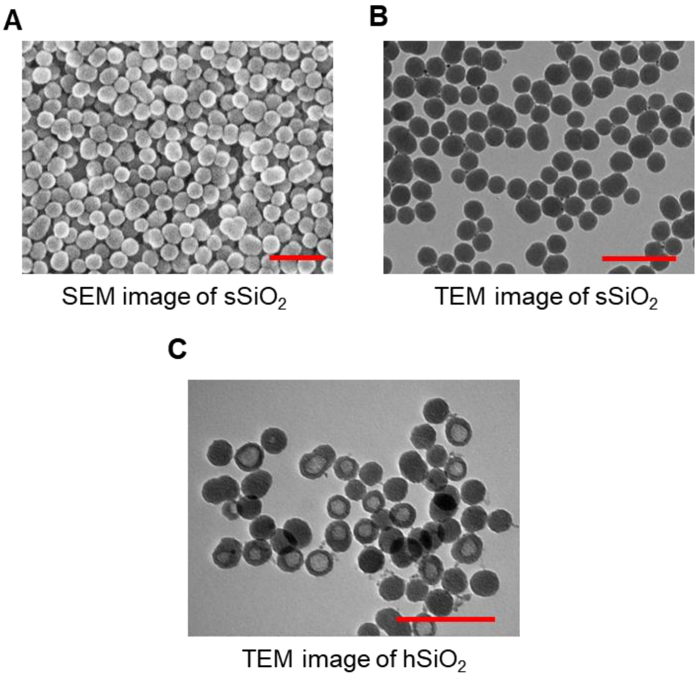


**Figure S11.** Topographic characterizations of $sSiO_2$ and $hSiO_2$. A) SEM and B) TEM image of $sSiO_2$, C) TEM image of $hSiO_2$, scale bar = 200 nm.

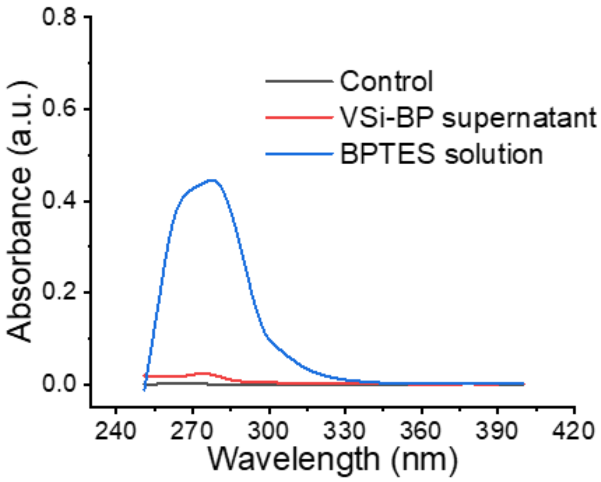


**Figure S12.** UV-vis absorption curves of deionized water, BPTES solution, and supernatant of VSi-BP after centrifugation (12,000 rpm, 15min). The BP loading ratio was calculated from the absorbance value at 278 nm, which is 94.68%.

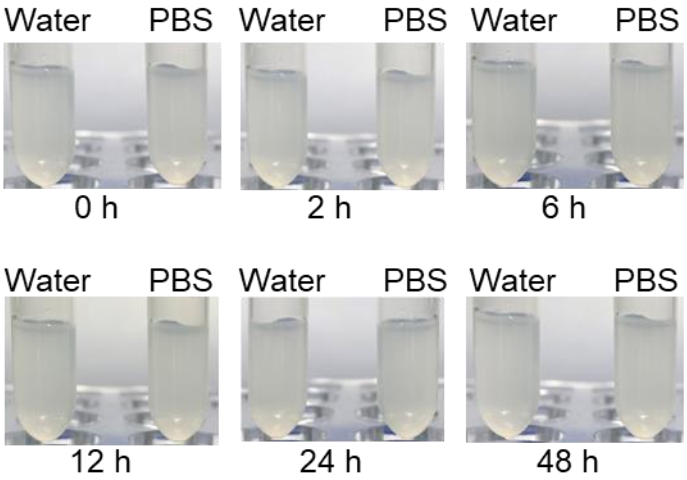


**Figure S13.** Images of nanoparticles immersed in deionized water and PBS for different times (0, 2, 6, 12, 24, 48h).

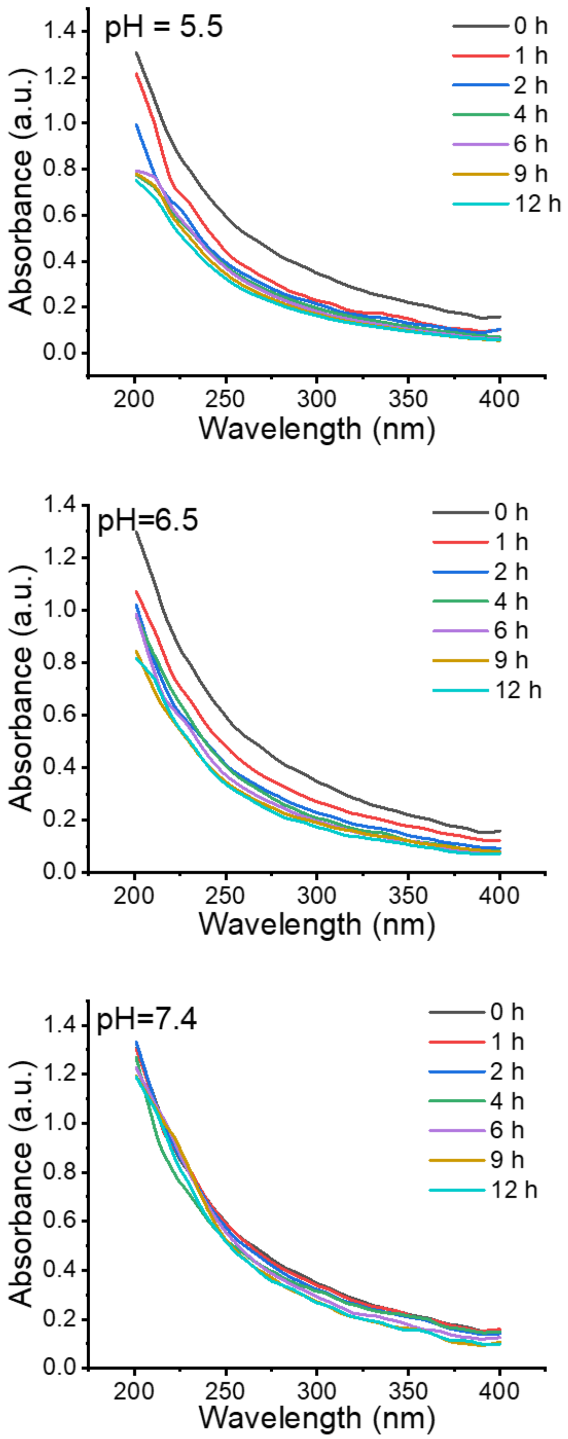


**Figure S14.** The degradation behavior of VSi@HA incubated in different conditions (pH 5.5, pH 6.5 and pH 7.4) measured by UV-vis spectra.

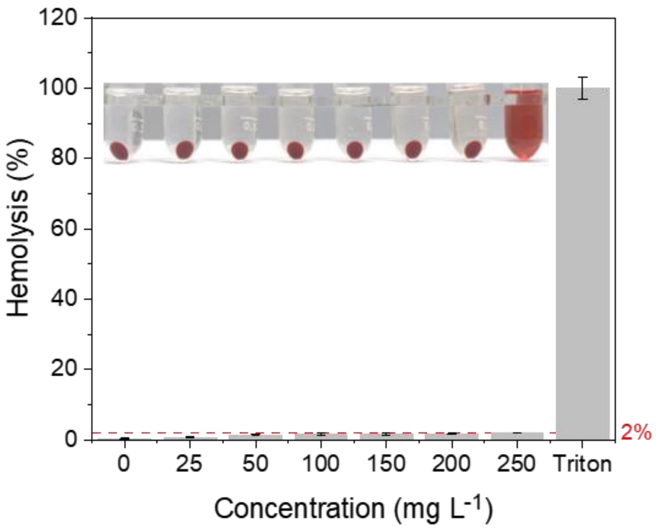


**Figure S15.** Images and hemolysis ratio of RBCs dispersed into PBS buffers containing different concentrations of VSi-BP@HA (0-250 mg $L^{-1}$) and Triton X-100 (0.4%) for 1h (*n* = 3). Data were represented as mean values ± SD.

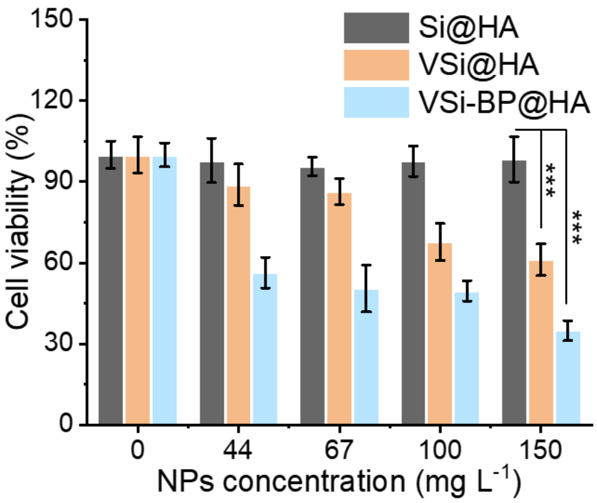


**Figure S16.** Cell viability of MC38 cells incubated with nanoparticles of various concentrations (0-150 mg $L^{-1}$) for 24h ($n$ = 8). Data were represented as mean values ± SD. and statistically calculated by one-way ANOVA. *$p < 0.05$, **$p < 0.01$, ***$p < 0.001$.

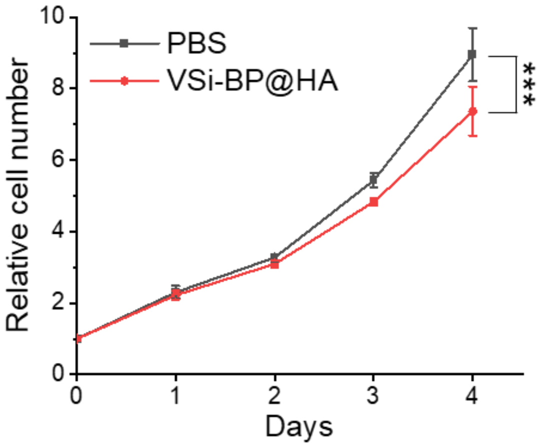


**Figure S17.** Effect of VSi-BP@HA treatment on the proliferation of 3T3 cells ($n$ = 8). Data were represented as mean values ± SD. and statistically calculated by two-tailed unpaired student's t-test. *$p$ < 0.05, **$p$ < 0.01, ***$p$ < 0.001.

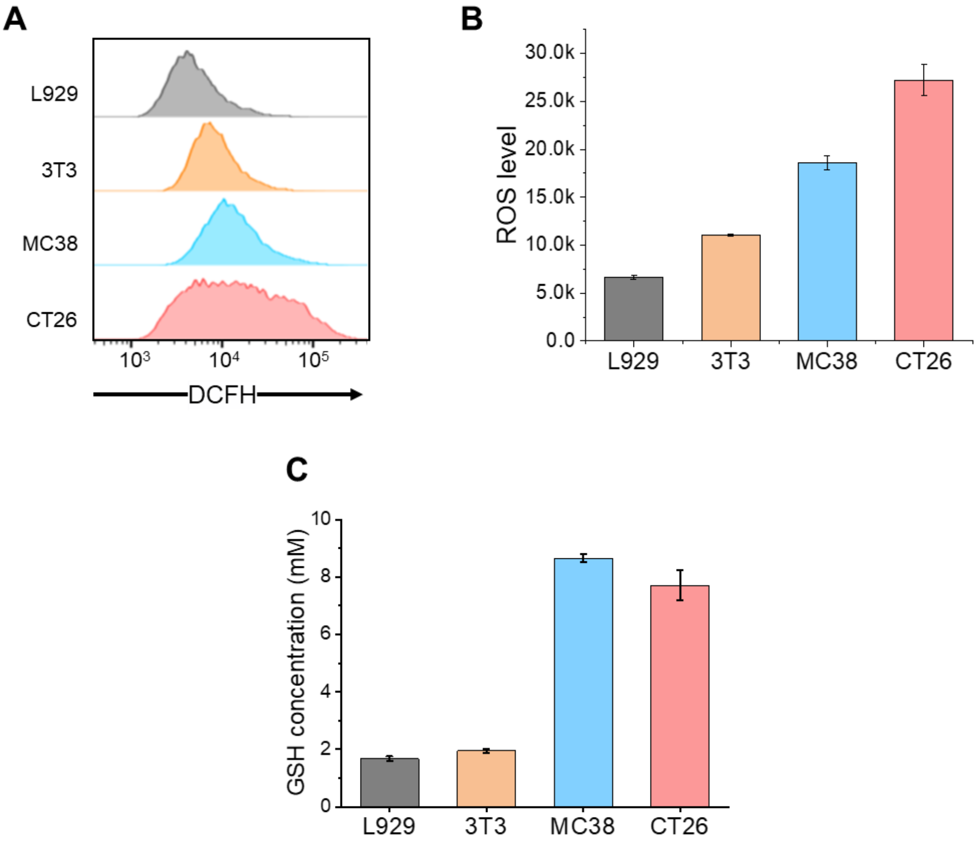


**Figure S18.** The ROS level and GSH concentration of different cells. The ROS level of L929, 3T3, MC38, and CT26 cells measured by A) flow cytometry and B) the corresponding quantization results. C) The concentration of GSH in L929, 3T3, MC38 and CT26 cells ($n$ = 3). Data were represented as mean values ± SD.

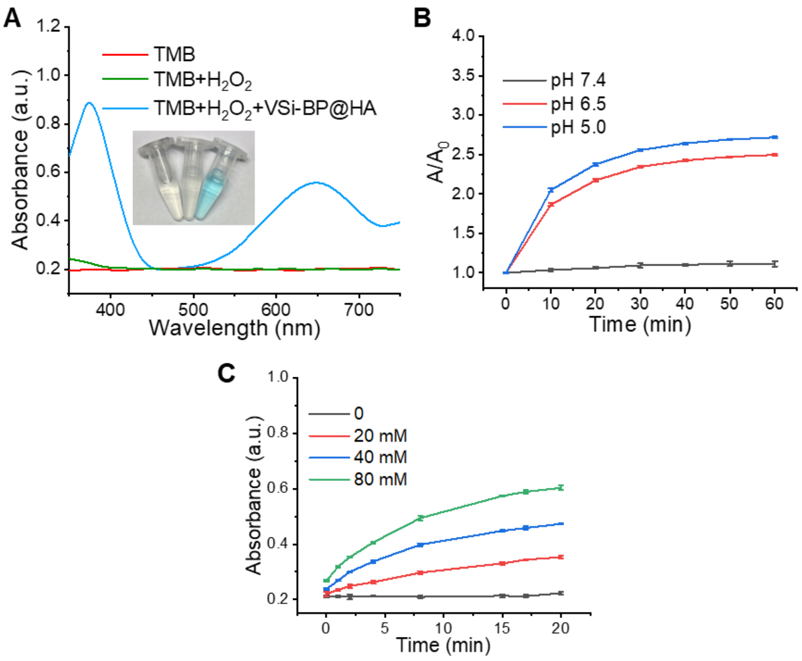


**Figure S19.** A) The Uv-vis spectra of TMB in different solutions at pH 6.5, $H_2O_2$: 80 mM, VSi-BP@HA: 100 µg $mL^{-1}$. The inset is the corresponding color change. B) Relative absorbance in 652 nm after VSi-BP@HA (100 µg $mL^{-1}$) incubation with TMB (1 mM) containing 80 mM $H_2O_2$ at different pH (7.4, 6.5, and 5.0). C) The absorbance curves of VSi-BP@HA (100 µg $mL^{-1}$) and TMB (1 mM) reactions for different times at various $H_2O_2$ concentrations (0, 20, 40, and 80 mM).

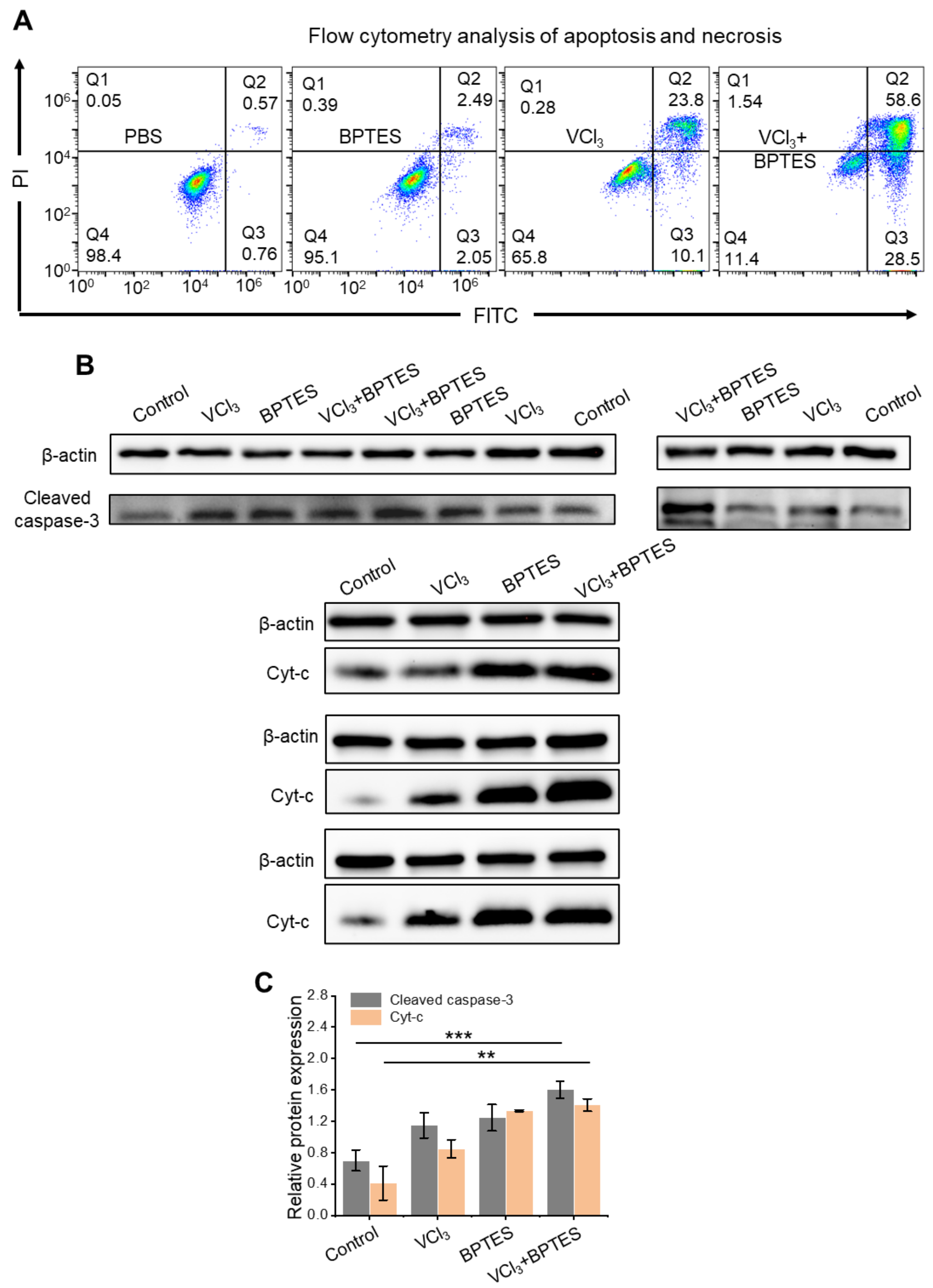


**Figure S20.** Evaluation of apoptosis of CT26 with different treatments. A) Evaluation of apoptosis of CT26 incubated with PBS, BPTES (4 μM), $VCl_3$ (36 μM), and BPTES + $VCl_3$ for 24h by flow cytometry; B) Western blotting analysis of cleaved caspase-3 and cytochrome c (cyt-c) in CT26 cells treated with PBS, BPTES (8 μM), $VCl_3$ (18 μM), and BPTES + $VCl_3$ for 24h. C) Semi-quantitative plot of B) (n = 3). Data were represented as mean values ± SD. and statistically calculated by the two-tailed unpaired student's t-test. *$p < 0.05$, **$p < 0.01$, ***$p < 0.001$.

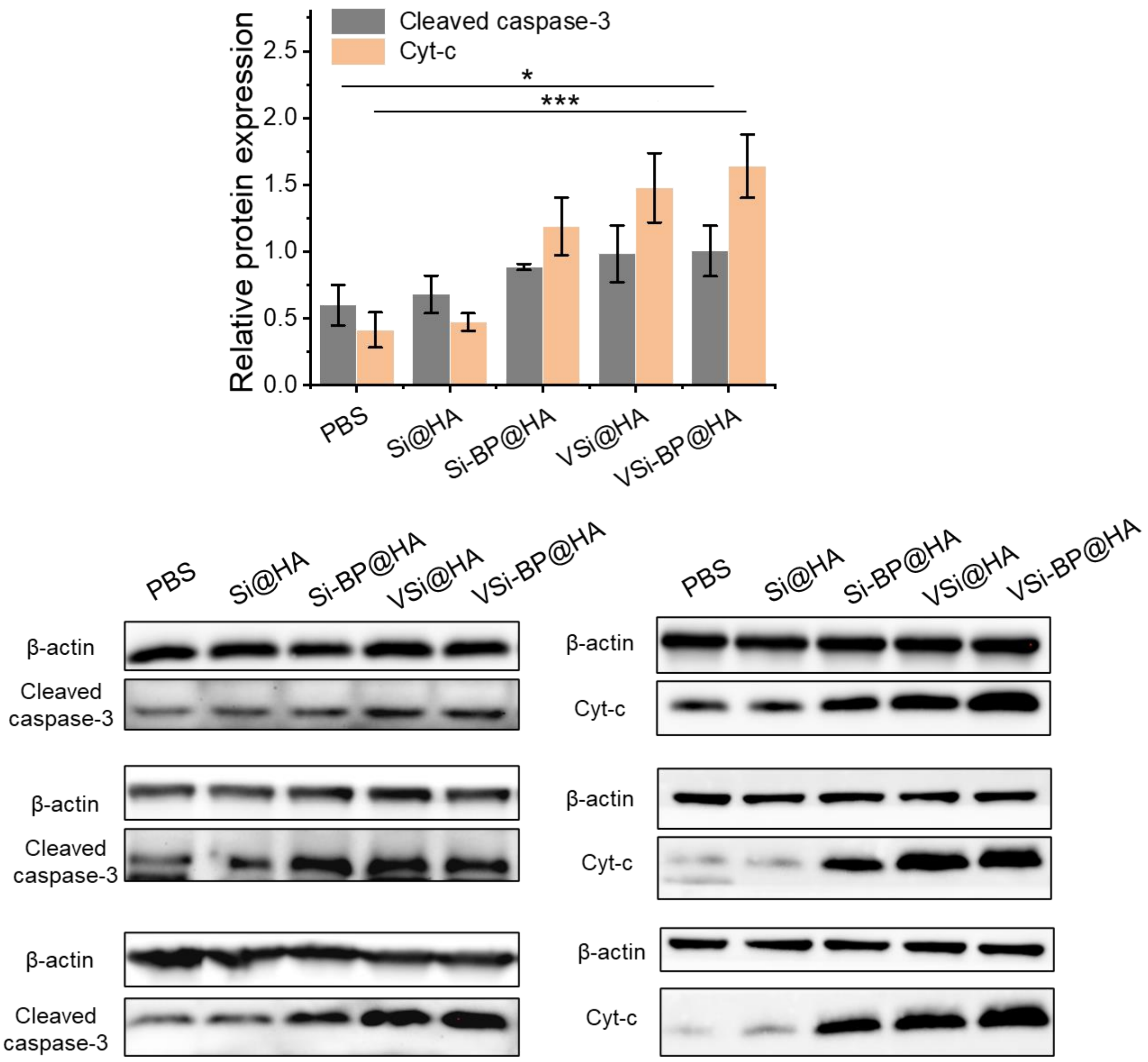


**Figure S21.** The expression of cleaved caspase-3 and cytochrome c (cyt-c) after nanoparticle treatment with CT26 (n = 3). Data were represented as mean values ± SD. and statistically calculated by the two-tailed unpaired student's t-test. $^{*}p < 0.05$, $^{**}p < 0.01$, $^{***}p < 0.001$.

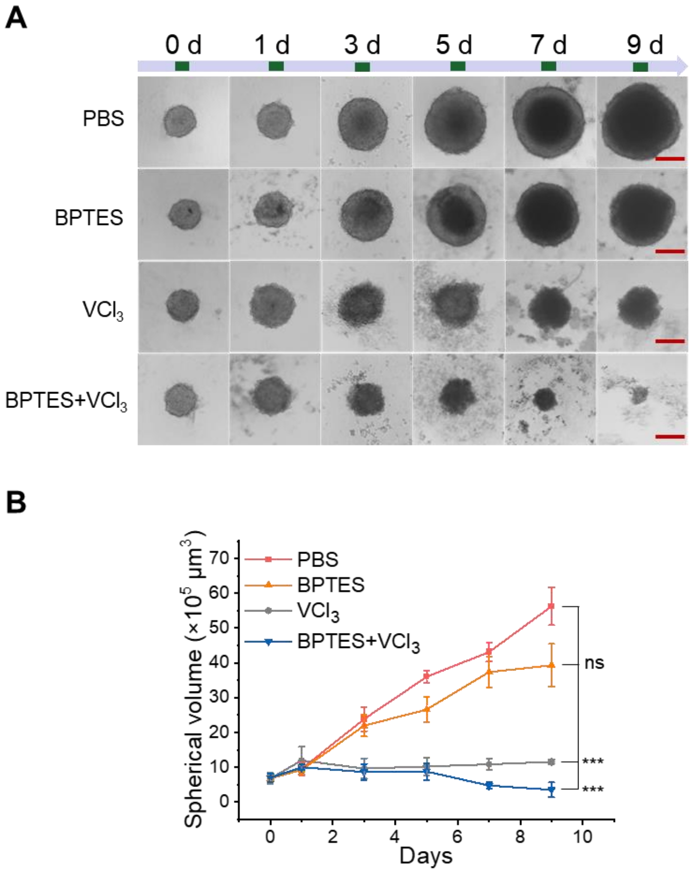


**Figure S22.** Effect of BPTES and $VCl_3$ on CT26 tumor spheroids. A) Images of CT26 tumor spheroids treated with BPTES (8 μM), $VCl_3$ (36 μM), BPTES+$VCl_3$. B) Spherical volume curves of A). The medium containing regents were refreshed every 2 days for 5 times. Scale bar = 200 μm ($n$ = 8). Data were represented as mean values ± SD. and statistically calculated by one-way ANOVA. *$p$ < 0.05, **$p$ < 0.01, ***$p$ < 0.001.

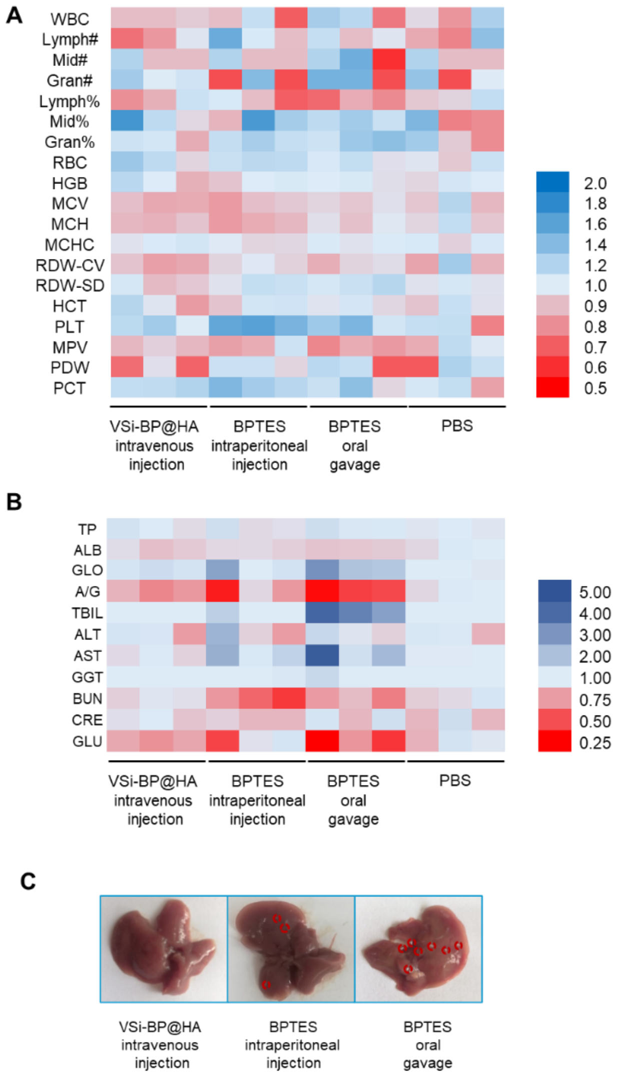


**Figure S23.** Biosafety of different drug delivery modes. A) Blood routine examination of the mice with different treatments. B) Blood biochemistry examination of BALB/C mice with different treatments. The modest alterations in blood chemistry parameters following VSi-BP@HA treatment may be attributed to its minor accumulation in the liver. C) Images of the mouse livers (white deposits circled in red) after administration of BPTES in different ways (intravenous injection, intraperitoneal injection, oral gavage). WBC, white blood cell; Lymph, lymphocyte; Mid, intermediate cell; Gran, granulocyte; Lymph%, lymphocyte%; Mid%, Intermediate cell%; Gran%, granulocyte%; RBC, red blood cell; HGB, hemoglobin; MCV, mean corpuscular volume; MCH, mean corpuscular hemoglobin; MCHC, mean corpuscular hemoglobin concentration; RDW-CV, red blood cell distribution width-coefficient of variation; RDW-SD, red blood cell distribution width-standard deviation; HCT, hematocrit; PLT, platelet count; MPV, mean platelet volume; PDW, platelet distribution width; PCT, plateletcrit.

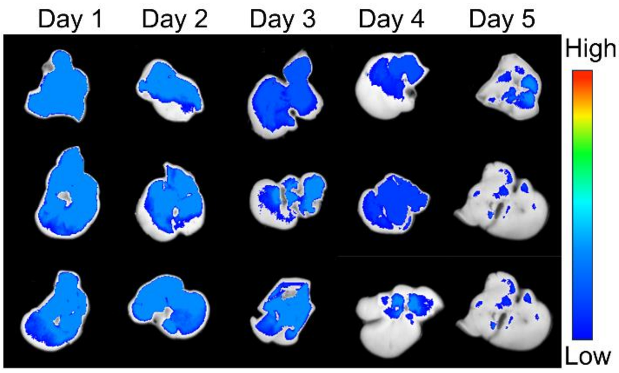


**Figure S24.** *Ex vivo* fluorescence images of the excised liver.

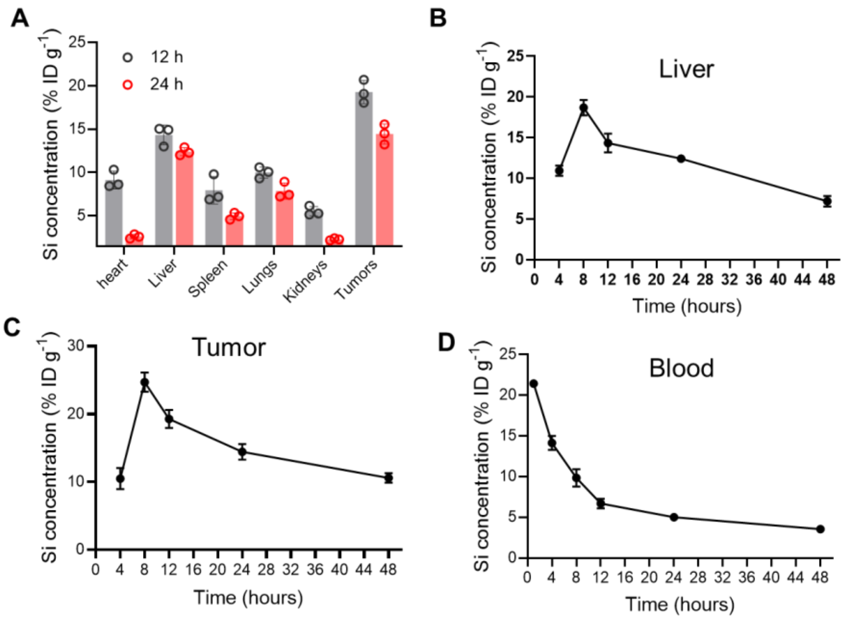


**Figure S25.** The metabolic behavior of VSi-BP@HA *in vivo* was assessed by quantifying the amount of Si present in various tissues using ICP-MS (n = 3). A) Distribution of the Si in various tissues of the body at 24h and 48h. B) Changes in Si content in livers. C) Changes in Si content in tumors. D) Changes in Si content in the blood.

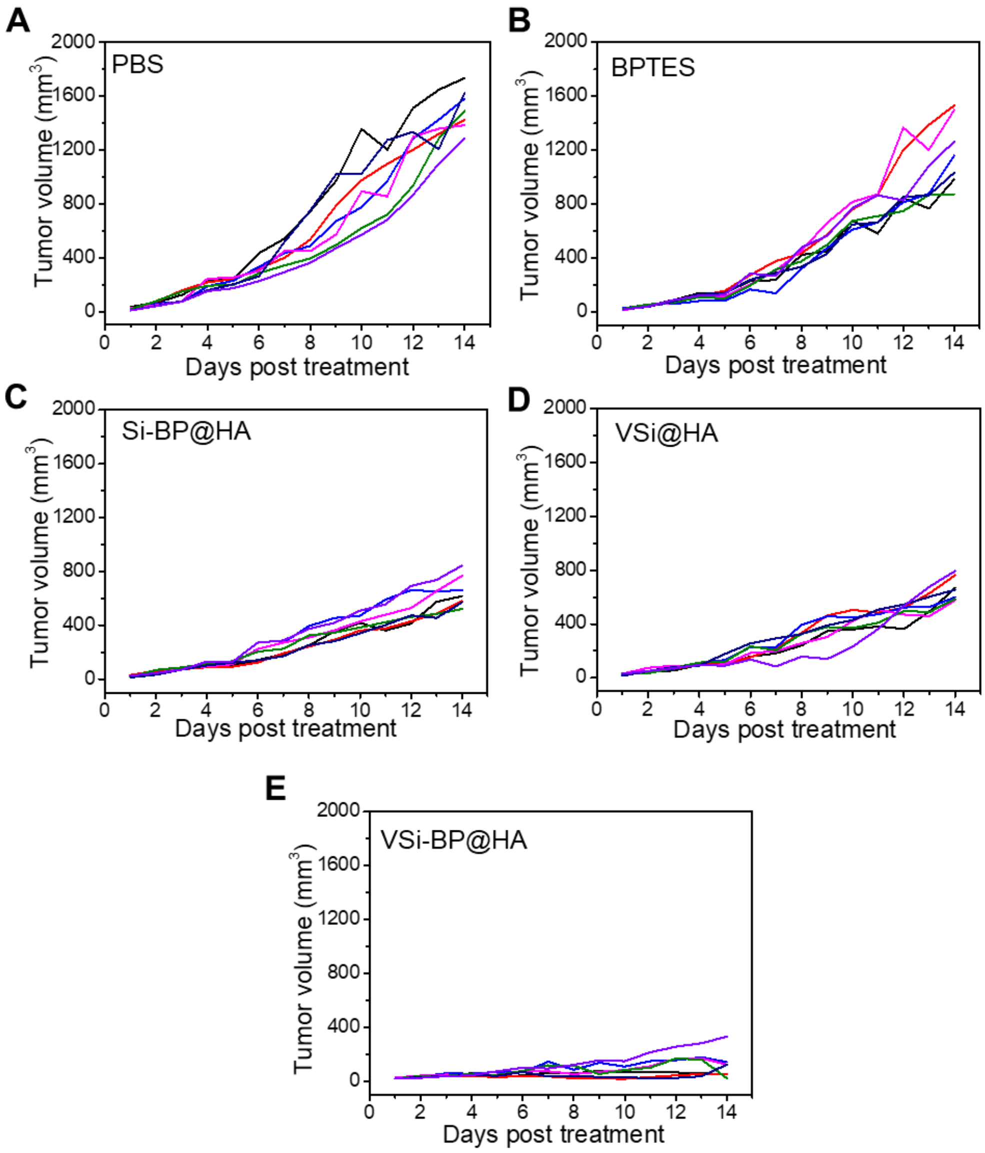


**Figure S26.** Tumor volume curves of different groups. Tumor volume curves of A) PBS, B) BPTES, C) Si-BP@HA, D) VSi@HA, E) VSi-BP@HA treated mice.

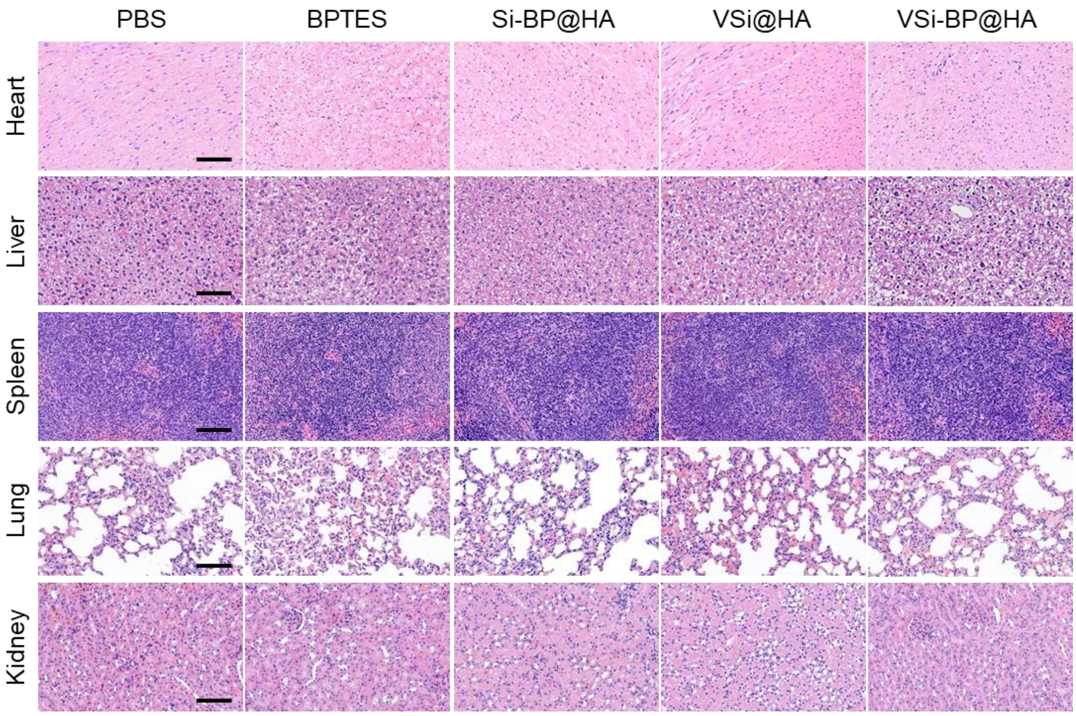


**Figure S27.** H&E-stained sections of major organs (heart, liver, spleen, lung and kidney). Scale bar = 100 μm.

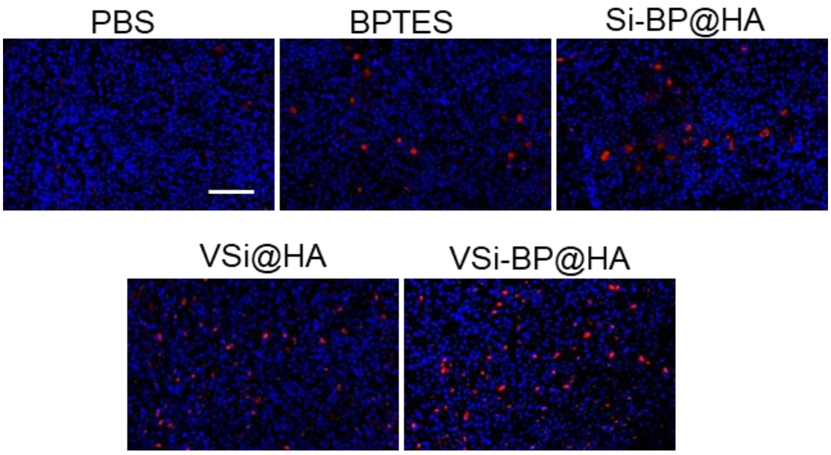


**Figure S28.** Cleaved caspase-3 fluorescence-stained sections of tumor tissue of mice treated with PBS, BPTES, Si-BP@HA, VSi@HA, VSi-BP@HA. Scale bar = 100 μm.

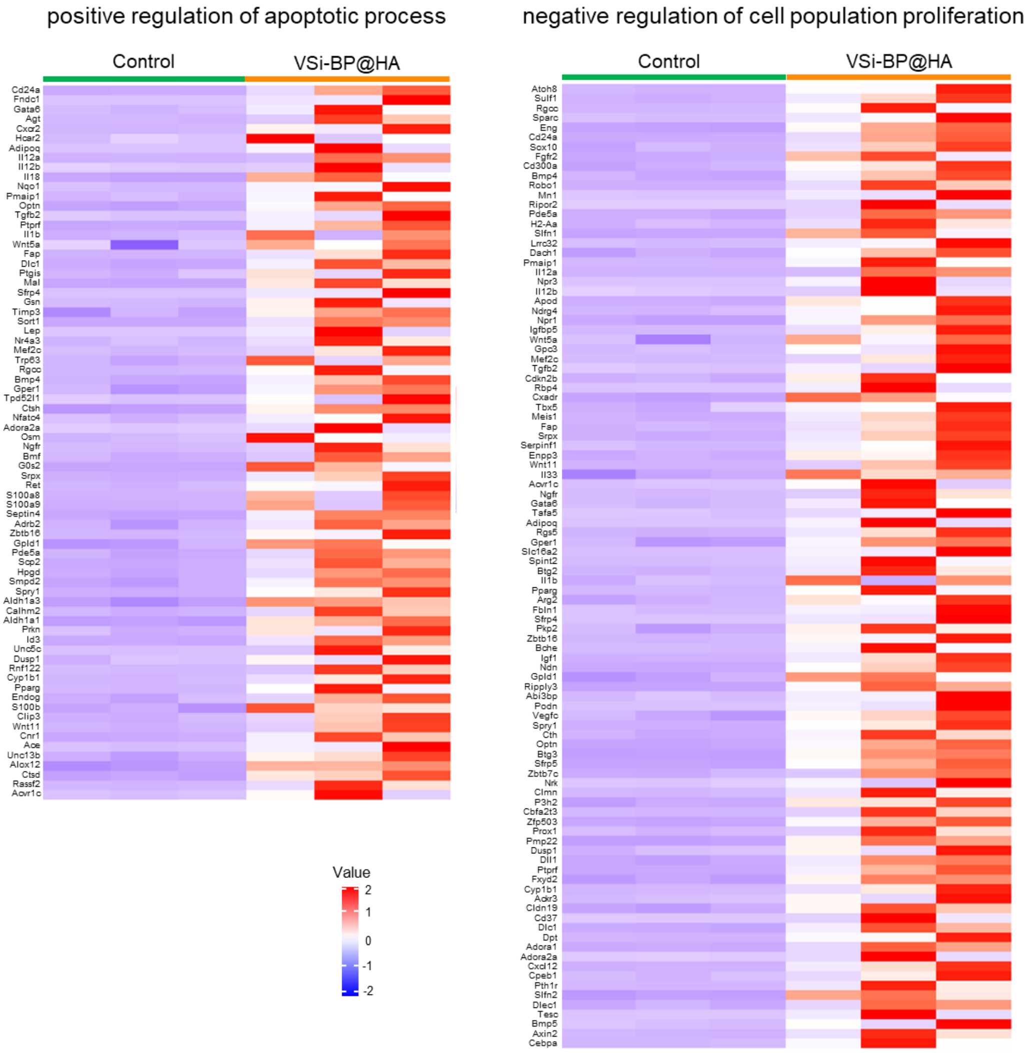


**Figure S29.** Heat-map resulting from analysis of DEGs related to "positive regulation of apoptotic process" and "negative regulation of cell population proliferation".

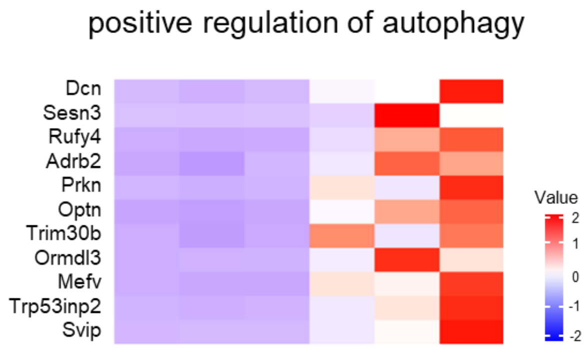


**Figure S30.** Heat-map resulting from analysis of DEGs related to “positive regulation of autophagy”.

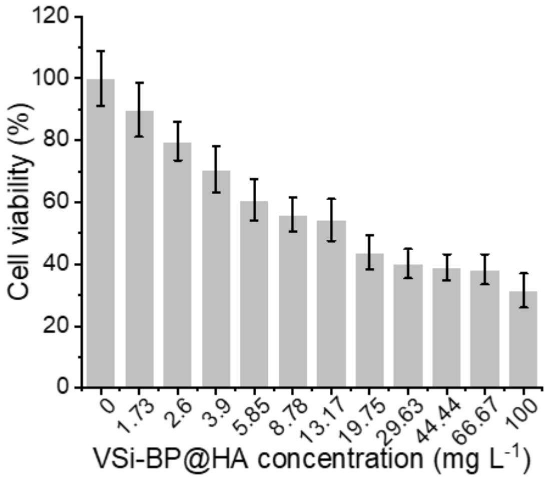


**Figure S31.** Cell viability of VSi-BP@HA-treated HCT116/L-OHP cells ($n$ = 8). Data were represented as mean values ± SD.

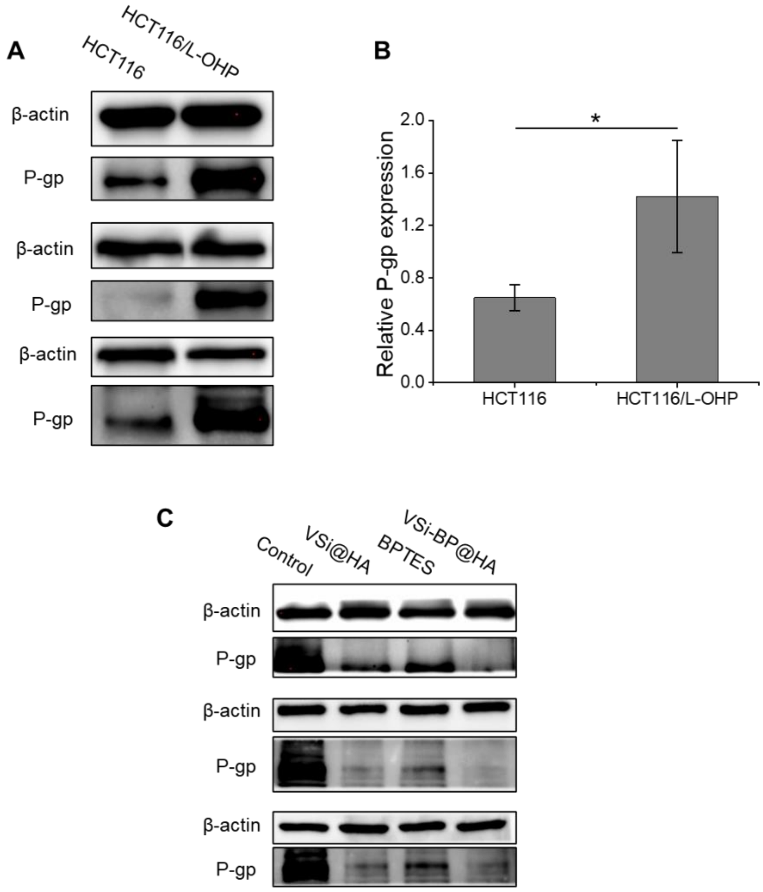


**Figure S32.** A) Western blotting analysis of P-gp in HCT116 cells and HCT116/L-OHP cells. B) Corresponding semi-quantitative plot of A). C) Western blotting analysis of P-gp expression in HCT116/L-OHP cells treated with BPTES (8 μM) or different nanoparticles (n = 3). Data were represented as mean values ± SD. and statistically calculated by the two-tailed unpaired student's t-test. *$p < 0.05$, **$p < 0.01$, ***$p < 0.001$.

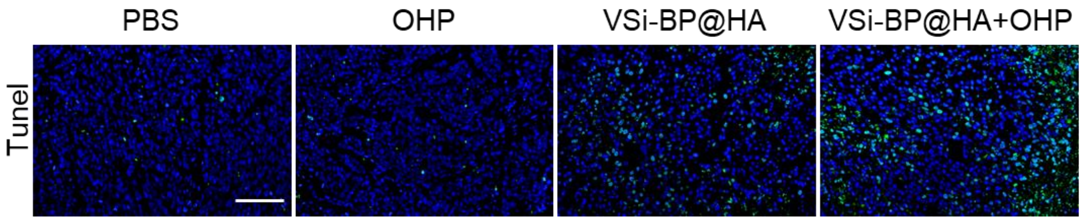


**Figure S33.** Representative TUNEL immunofluorescence staining images of tumor slices obtained at the end of the experiments. Scale bar = 100 μm.

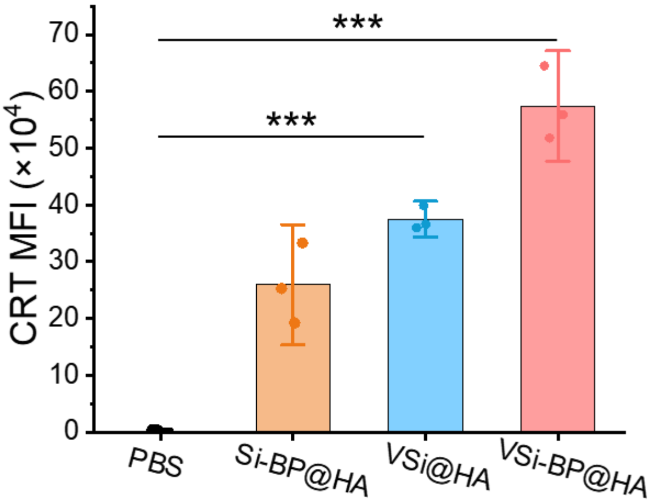


**Figure S34.** The corresponding mean fluorescence intensity of flow cytometry analysis of CRT in Figure 5D ($n$ = 3). Data were represented as mean values ± SD. and statistically calculated by one-way ANOVA. *$p < 0.05$, **$p < 0.01$, ***$p < 0.001$. CRT, Calreticulin.

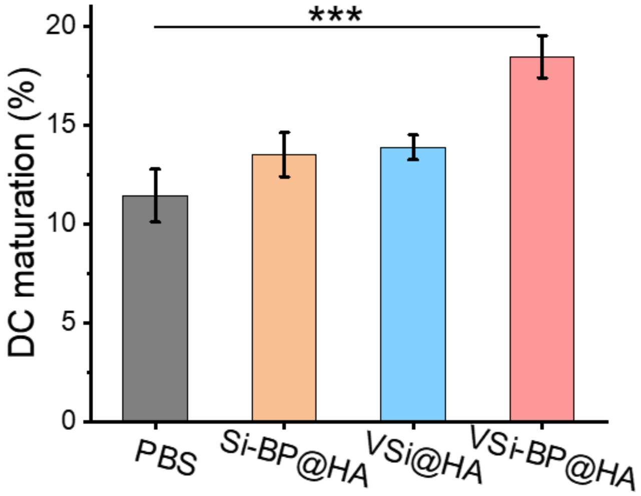


**Figure S35.** The DC maturation detected by flow cytometry (*n* = 3). Data were represented as mean values ± SD. and statistically calculated by one-way ANOVA. **p* < 0.05, ***p* < 0.01, ****p* < 0.001.

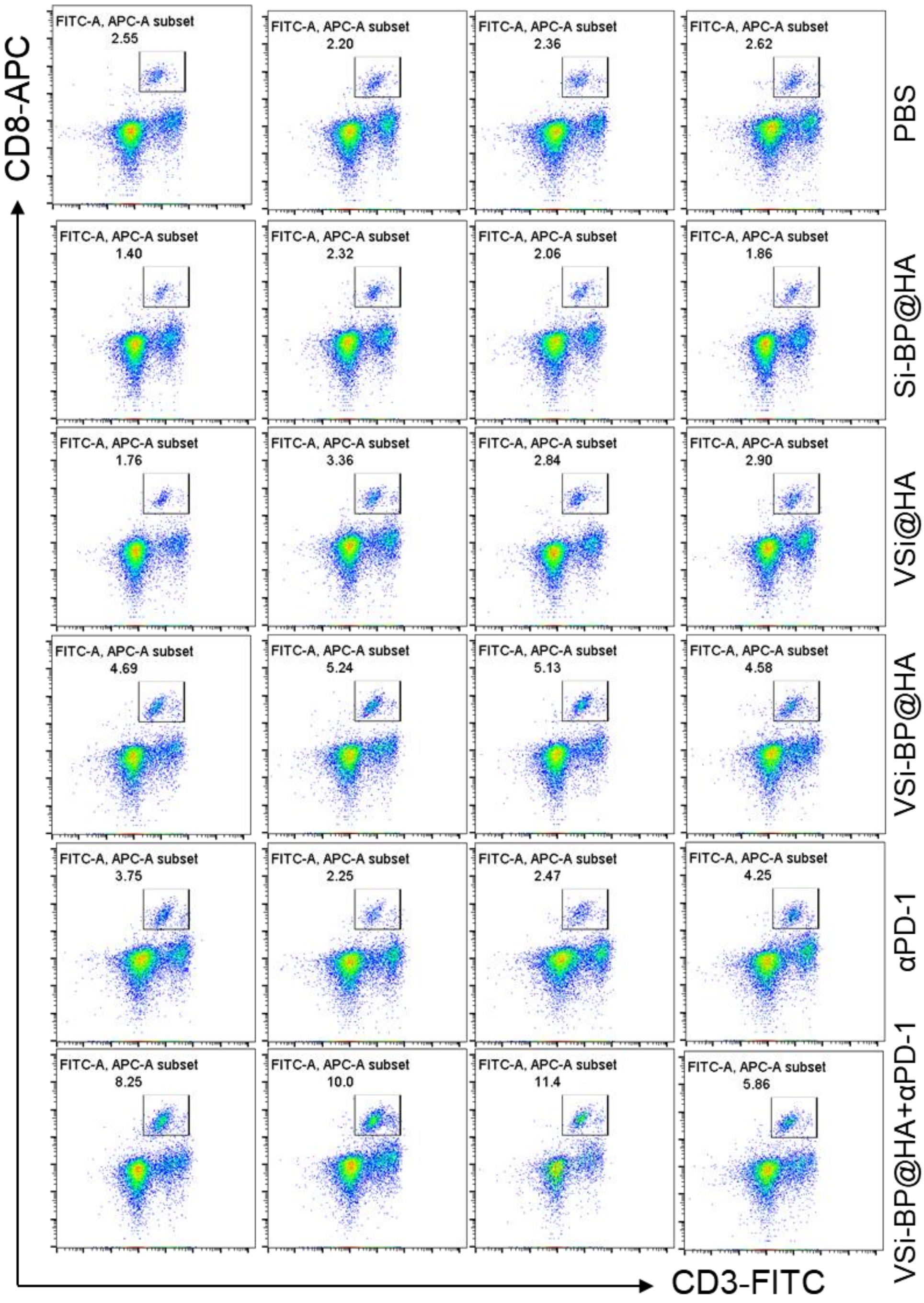


**Figure S36.** Flow cytometric data of $CD3^+CD8^+$ T cells in tumors.

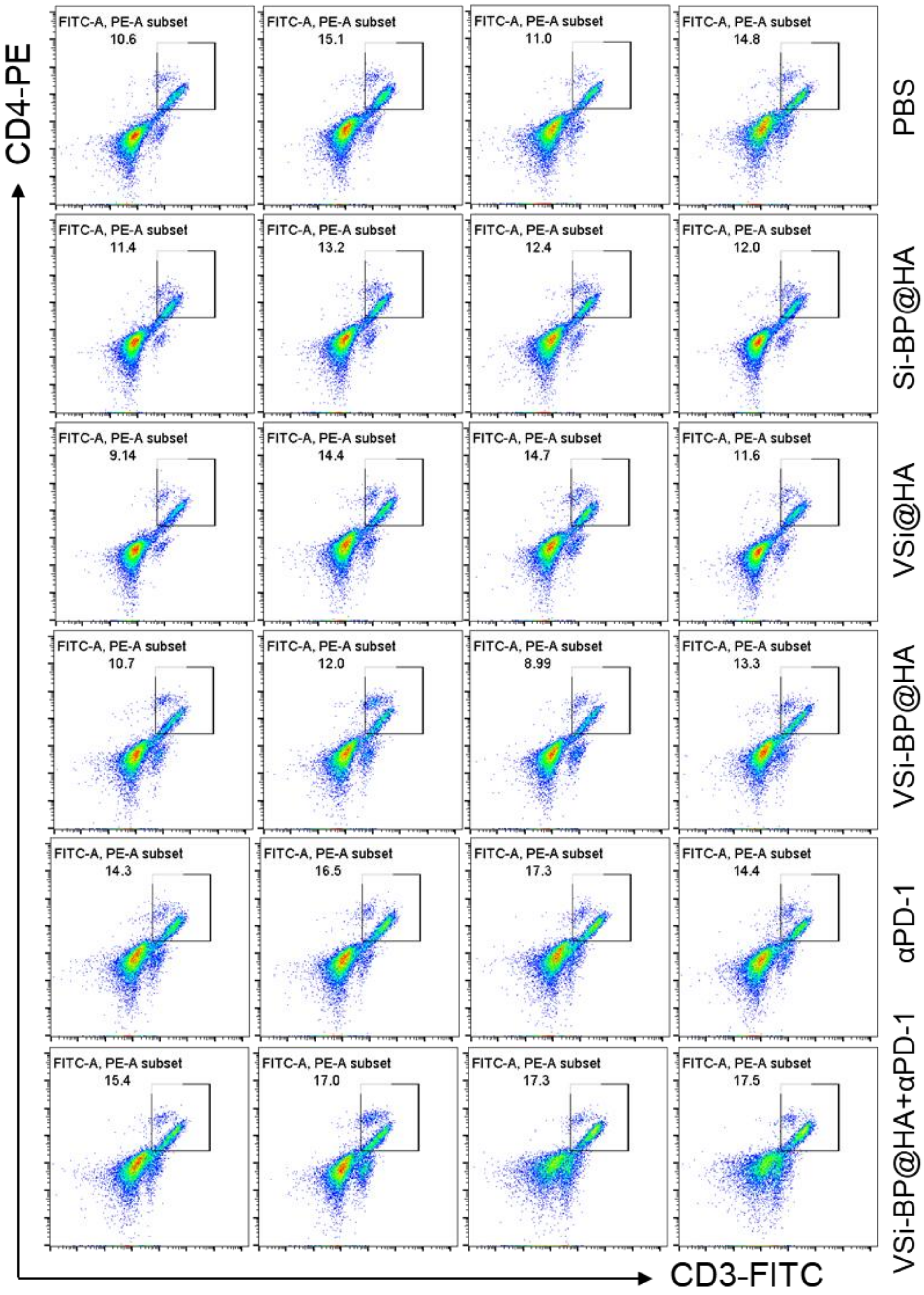


**Figure S37.** Flow cytometric data of $CD3^{+}CD4^{+}$ T cells in tumors.

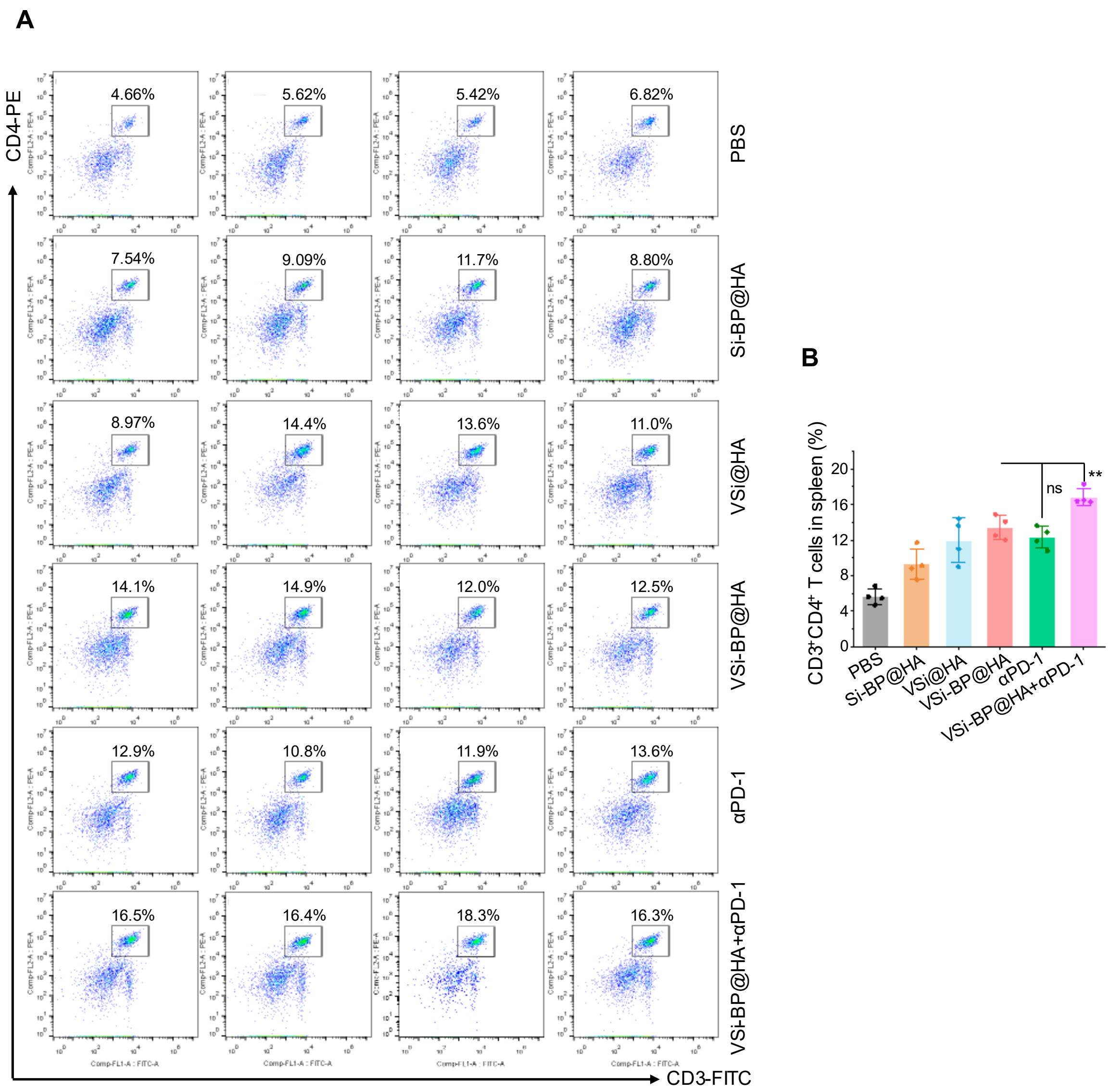


**Figure S38.** A) Flow cytometric data of CD3$^{+}$CD4$^{+}$ T cells in spleens and B) corresponding semi-quantification of A).

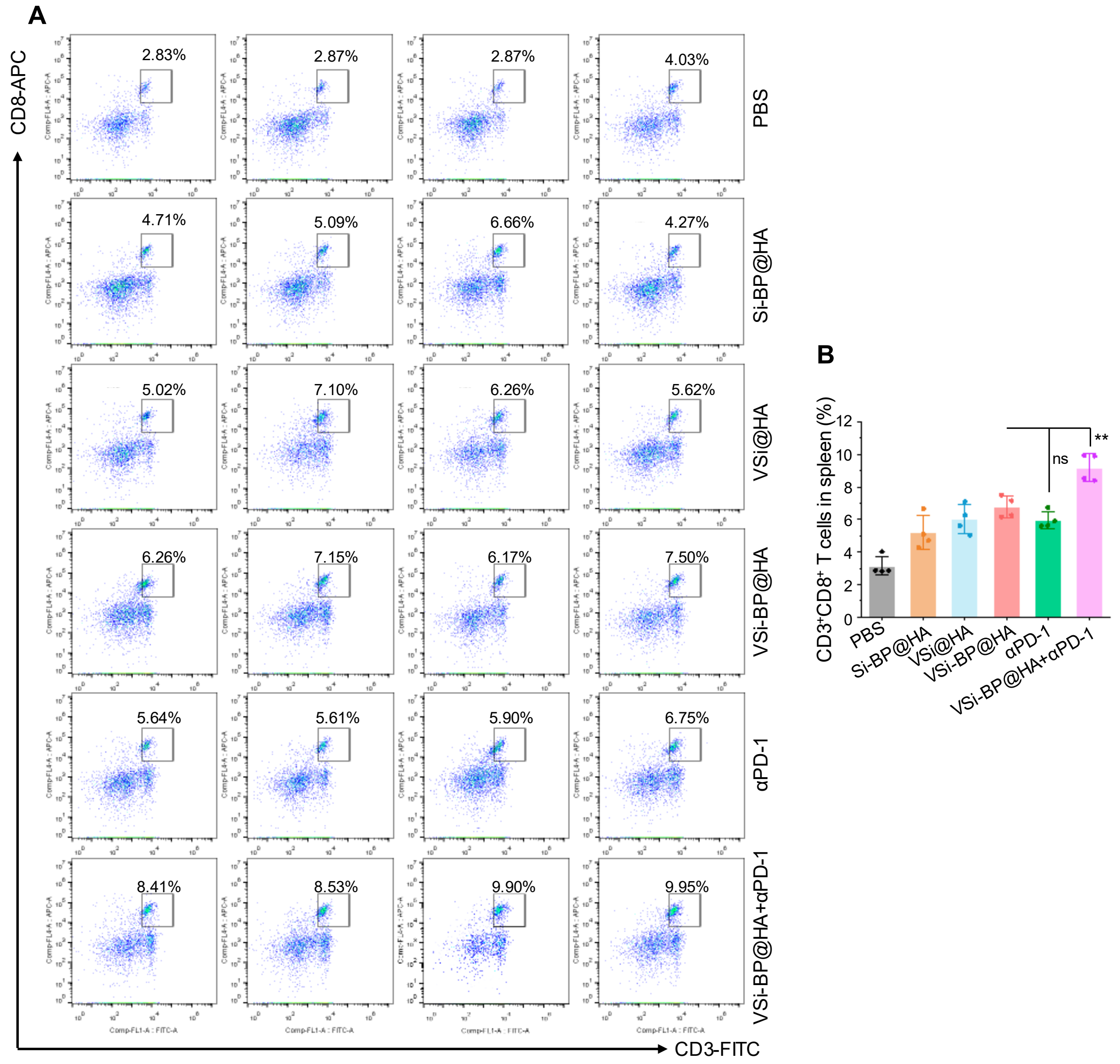


**Figure S39.** A) Flow cytometric data of CD3$^+$CD8$^+$ T cells in spleens and B) corresponding semi-quantification of A).

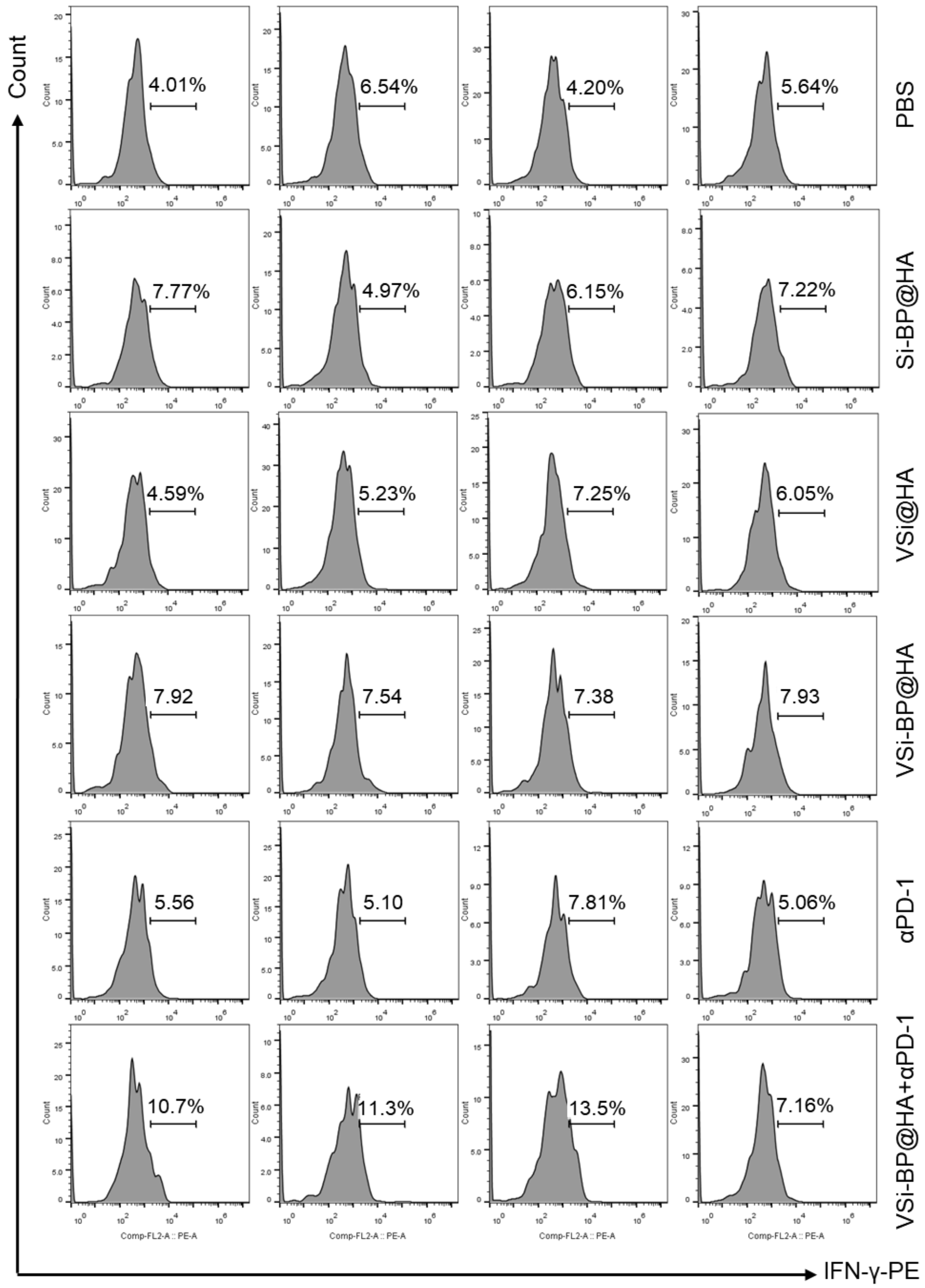


**Figure S40.** Flow cytometric data of $CD3^{+}CD8^{+}IFN\text{-}\gamma^{+}$ T cells in spleens.
